# hUC-MSCs-derived MFGE8 ameliorates locomotor dysfunction via inhibition of ITGB3/ NF-κB signaling in an NMO mouse model

**DOI:** 10.1038/s41536-024-00349-z

**Published:** 2024-01-20

**Authors:** Huiming Xu, Wei Jiang, Xuejia Li, Jiaohua Jiang, Shabbir Khan Afridi, Longhui Deng, Rui Li, Ermei Luo, Zhaoqing Zhang, Yu-Wen Alvin Huang, Yaxiong Cui, Kwok-Fai So, Haijia Chen, Wei Qiu, Changyong Tang

**Affiliations:** 1 Department of Neurology, The Third Affiliated Hospital of Sun Yat-Sen University, 600 Tianhe Road, Guangzhou, 510630 Guangdong Province China; 2 Guangzhou SALIAI Stem Cell Science and Technology Co., Ltd., Guangdong Saliai Stem Cell Research Institute, Guangzhou, Guangdong Province China; 3 Faculty of Biological Sciences, School of Biomedical Sciences, University of Leeds, Leeds, LS2 9JT UK; 4 Department of Molecular Biology, Cell Biology, and Biochemistry, Brown University, 70 Ship 15 Street, Providence, RI 02903 USA; 5 State Key Laboratory of Membrane Biology, Tsinghua-Peking Center for Life Sciences, IDG/McGovern Institute for Brain Research, Beijing Advanced Innovation Center for Structural Biology, School of Pharmaceutical Sciences, Tsinghua University, Beijing, 100084 China

**Keywords:** Cell death in the nervous system, Neurological disorders

## Abstract

Neuromyelitis optica (NMO) is a severe autoimmune inflammatory disease of the central nervous system that affects motor function and causes relapsing disability. Human umbilical cord-derived mesenchymal stem cells (hUC-MSCs) have been used extensively in the treatment of various inflammatory diseases, due to their potent regulatory roles that can mitigate inflammation and repair damaged tissues. However, their use in NMO is currently limited, and the mechanism underlying the beneficial effects of hUC-MSCs on motor function in NMO remains unclear. In this study, we investigate the effects of hUC-MSCs on the recovery of motor function in an NMO systemic model. Our findings demonstrate that milk fat globule epidermal growth 8 (MFGE8), a key functional factor secreted by hUC-MSCs, plays a critical role in ameliorating motor impairments. We also elucidate that the MFGE8/Integrin αvβ3/NF-κB signaling pathway is partially responsible for structural and functional recovery, in addition to motor functional enhancements induced by hUC-MSC exposure. Taken together, these findings strongly support the involvement of MFGE8 in mediating hUC-MSCs-induced improvements in motor functional recovery in an NMO mouse model. In addition, this provides new insight on the therapeutic potential of hUC-MSCs and the mechanisms underlying their beneficial effects in NMO.

## Introduction

Neuromyelitis optica (NMO) are a group of autoimmune inflammatory demyelinating diseases that primarily affect the optic nerves, spinal cord, brainstem, and occasionally the cerebrum^1,2^. NMO is characterized by recurrent attacks of visual, motor, and/or sensory dysfunction, which can result in blindness, paraplegia, and even lethal outcome^2,3^. NMO pathology is associated with the presence of serum autoantibodies against for the water channel aquaporin 4 (AQP4), which is highly expressed on the membrane of astrocytic endfeet adjacent to microvessels at the blood–brain barrier (BBB)^4^. The binding of the NMO-IgG to the three-dimensional conformational epitopes in the extracellular loops of AQP4 on the surface of astrocytes may trigger the pathophysiology underlying NMO^5^. Despite the known interaction between NMO-IgG and AQP4, the mechanisms that trigger NMO lesion development and loss of function in patients remains unknown and contentious. Thus, advancing our understanding of NMO mechanisms is critical for improving the efficacy of current treatment strategies. Some promising currently available treatments for NMO include B-cell depletion (rituximab, inebilizumab), interleukin-6 signaling blockade (tocilizumab, satralizumab), and complement inhibition (eculizumab)^4,6^. However, some patients who receive these therapies still experience disease relapse. To date, the optimal first-line treatment to reduce relapse rate remains unclear. Therefore, there is a major need to explore alternative treatment options for this disease.

Human umbilical cord-derived mesenchymal stem cells (hUC-MSCs) are a polyclonal population of adult stromal cells that possess immunosuppressive and anti-inflammatory properties and are associated with several advantages, such as fewer ethical concerns, a less invasive isolation procedure, low immunogenicity, high proliferation capacity, and multi-lineage differentiation capability^7,8^. Due to these properties, hUC-MSCs can be used in the development of medical products, particularly for treating inflammatory or immune diseases, such as type I diabetes, systemic *lupus erythematosus* (SLE), multiple sclerosis (MS), rheumatoid arthritis (RA), Crohn’s disease, and allograft-related diseases^7,9,10^. However, the potential of hUC-MSCs as a treatment option for NMO remains to be explored.

Substantial evidence has indicated that hUC-MSCs exert their therapeutic effects primarily through paracrine pathways via the secretion of various neurotrophic and survival-growth factors, cytokines, chemokines, and other soluble and contact factors^11–13^. These paracrine factors contribute to anti-inflammatory and immune modulation, tissue remodeling, and cellular homeostasis during regeneration^14,15^. Therefore, there is a need to identify key functional factors secreted by hUC-MSCs that could play important roles in hUC-MSCs-modulated recovery of NMO lesions.

In the present study, we aimed to investigate the effects of hUC-MSCs on the recovery of motor dysfunction in an NMO systemic mouse model. Our results demonstrate that the core functional factor milk fat globule epidermal growth 8 (MFGE8), which is secreted by hUC-MSCs, plays a critical role in recovery of astrocyte activation and prevention of demyelination by downregulating inflammatory factors expression. Mechanistically, we show that the structural and functional recovery elicited by exposure to hUC-MSCs is at least partially mediated by MFGE8 through the inhibition of NF-κB transcriptional activation by the integrin αvβ3. Collectively, our study highlights a foundational molecular mechanism of the pleiotropic cytokine MFGE8, which underlined the hUC-MSCs-based cell therapy for NMO intervention.

## Results

### Immune-stimulated mouse primary astrocytes release NF-κB-Targeting pro-inflammatory factors

We first analyzed published datasets to characterize expression levels of pro-inflammatory cytokines in immune-stimulated primary mouse astrocytes^16^. There was an overlap of 26 pro-inflammatory cytokines out of 1729 upregulated differentially expressed genes (DEGs) in mouse primary astrocytes treated with human anti-AQP4 autoantibodies purified from NMO patients’ plasma (hsAQP4-IgG) (Supplementary Fig. 1a), the proper control conditions of human control IgG (hsCtrl-IgG, pooled from healthy subject’s plasma), recognized a group of C-C and C-X-C motif chemokine genes, including CCL2, 7, 3, 9, 4, 6 and CXCL 1, 9, 10, 5, 11, 3, 16, 12, 13, together with TNF-α, IL-6, IL-1α, IL-1β (Supplementary Fig. 1b).

**Fig. 1:**
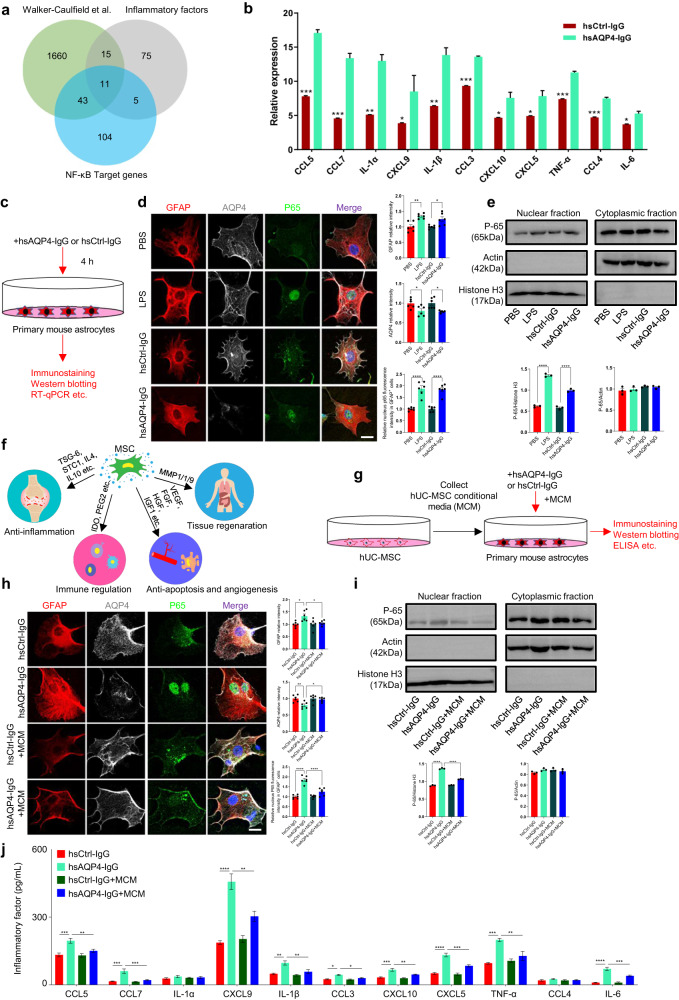
hUC-MSC conditional medium inhibits astrocytopathy and NF-κB signaling activation in vitro. **a** Venn diagram showing the overlap of differentially expressed genes (DEGs) identified by transcriptomic analysis of mouse astrocytes stimulated with human anti-AQP4 antibodies (hsAQP4-IgG) or control human IgG (hsCtrl-IgG). The diagram shows that 11 out of 26 pro-inflammatory cytokines that were upregulated by hsAQP4-IgG were NF-κB target genes. The green portion represents the results of Walker-Caulfield et al.^16^, the gray portion represents the pro-inflammatory database, and the blue portion represents the NF-κB target genes database. **b** Relative levels of the 11 overlapped genes in primary astrocytes treated with hsAQP4-IgG and hsCtrl-IgG from the published dataset by Walker-Caufiled et al. ^16^. **c** Schematic diagram of the experimental design, showing treatment of primary cultures of wild-type mouse astrocytes with LPS, hsAQP4-IgG, hsCtrl-IgG, or control PBS, followed by Immunostaining, Western blotting, and RT-qPCR. **d** Representative confocal images (left panels) and quantification of fluorescence signals (right bar graphs) of GFAP (red), AQP4 (gray), NF-κB-P65 (green), and DAPI (blue) in primary mouse astrocytes treated with LPS, hsAQP4-IgG, hsCtrl-IgG, or PBS. Error bars represent SEM. *n* = 6 experiments per group. Scale bar, 20 µm. **e** Immunoblotting analysis of cytoplasmic and nuclear fractions of primary mouse astrocytes treated with hsAQP4-IgG or hsCtrl-IgG, followed by treatment with hUC-MSCs conditional media (MCM). The densitometric quantifications (lower bar graphs) revealed that MCM abrogated hsAQP4-IgG-induced accumulation of active NF-κB-P65 in the cell nucleus. *n* = 3 experiments per group. **f** Cartoon image showing the mechanism of hUC-MSCs therapeutic activity, promoting anti-inflammatory responses, anti-apoptosis and regeneration of injured sites. **g** Schematic diagram of experiments assessing the effect of hUC-MSCs-secreted factors on primary mouse astrocytes treated with hsAQP4-IgG or hsCtrl-IgG, followed by immunostaining, western blotting, and ELISA analysis. **h** Representative confocal images (upper panels) and quantification of fluorescence signals (lower bar graphs) of GFAP, AQP4, and NF-κB in primary mouse astrocytes treated with hsAQP4-IgG or hsCtrl-IgG, followed by treatment with hUC-MSCs conditional media (MCM). *n* = 6 experiments per group. Scale bar, 20 µm. **i** The primary mouse astrocytes were treated with hsAQP4-IgG or hsCtrl-IgG, followed by treatment of hUC-MSCs conditional media (MCM) for 4 hours. Then cytoplasmic fraction and nuclear fraction were lysed and harvested at the same time. The treated astrocyte lysates were then subject to immunoblotting for assaying the protein levels of NF-κB-P65 and the control β-actin or Histone H3. The densitometric quantifications (low bar graphs) revealed that the MCM abrogated the role of hsAQP4-IgG induced accumulation of active NFκB-P65 into the cell nuclear. **j** Bar graph showing that hUC-MSCs media downregulated NF-κB target pro-inflammatory factor expression by ELISA tests. (*n* = 4 experiments/group). Statistical analysis was performed using Student’s t-test for (**b**) and one-way ANOVA followed by Tukey’s post hoc multiple comparisons test for **d**, **e**, **h**–**j**. Non-significant comparisons are not identified. *p < 0.05, **p < 0.01, ***p < 0.001, ****p < 0.0001. AQP4 aquaporin protein4, LPS lipopolysaccharide, NMO neuromyelitis optica, PBS phosphate buffer saline, MCM MSCs-conditioned media, hUC-MSCs human umbilical cord mesenchymal stem cells, ELISA enzyme-linked immunosorbent assay.

In addition, we used the computational tool Pathview to integrate and extract KEGG pathway-based data, which revealed specific enrichment of DEGs in the NF-κB pathway (Supplementary Fig. 1c), known to be critical for neurotoxic inflammation of astrocytes and activated by hsAQP4-IgG. Our analysis showed an overlap of eleven NF-κB target genes, including CCLs 5, 7, 3, 4, CXCLs 9, 10, 5, IL-1α, IL-1β, TNF-α, and IL-6, out of 26 pro-inflammatory cytokines significantly induced with hsAQP4-IgG treatment in cultured mouse primary astrocytes (Fig. 1a, b). Next, we used previous studies to determine levels of the eleven pro-inflammatory factors in NMO patients. Seven of these cytokines (including CXCL10, CCL4, IL-6, TNF-a, CXCL5, IL-1β, and CCL3) were significantly increased in NMO patients compared with healthy controls (HCs) (Supplementary Table 1). To further validate these conclusions, we cultured primary astrocytes from neonatal mice as described previously^17,18^ and treated them with LPS (100 ng/mL), hsAQP4-IgG (100 ng/mL), with proper control conditions of vehicle (PBS), and hsCtrl-IgG (100 ng/mL) for 4 hours (Fig. 1c). Immunofluorescence analysis showed that treatment with hsAQP4-IgG, but not hsCtrl-IgG or LPS, lead to the internalization and degradation of AQP4 in astrocytes (Fig. 1d), which is a hallmark of NMO pathology that distinguishes NMO from the autoimmune disease multiple sclerosis^19^. We also observed a high level of NF-κB-P65 translocation in nucleus from hsAQP4-IgG or LPS-treated astrocytes relative to control astrocytes (Fig. 1d). These results were also confirmed in a Western blotting analysis, we treated primary mouse astrocytes with hsAQP4-IgG (or hsCtrl-IgG), LPS (or PBS) for 4 hours, and then lysed the cytoplasmic fraction and nuclear fraction, and harvested at the same time. The treated astrocyte lysates were then subjected to immunoblotting to assay protein levels of NF-κB-P65 and the controls β-actin (cytoplasmic protein) or histone H3 (nuclear protein). Quantitative analysis revealed that hsAQP4-IgG treatment induced a rapid accumulation of active NF-κB-P65 in the cell nucleus (Fig. 1e). Taken together, these findings indicate that NF-κB activation can be triggered by hsAQP4-IgG stimuli in mouse primary astrocytes.

Next, we examined whether NF-κB targeted proinflammatory factor were also induced by hsAQP4-IgG in astrocytes. We found that the eleven representative NF-κB target pro-inflammatory genes were strongly induced in hsAQP4-IgG group by quantitative reverse transcription polymerase chain reaction (RT-qPCR) analysis (Supplementary Fig. 1d), in contrast to the hsCtrl-IgG groups. This indicates that hsAQP4-IgG-treated astrocytes exhibit constitutive NF-κB activation and release many pro-inflammatory cytokines.

### In vitro inhibition of immune-induced astrocyte activation and pro-inflammatory factor release by hUC-MSCs through secretion

The NF-κB pathway has been widely recognized to play a critical role in immune-mediated inflammatory diseases, including rheumatoid arthritis^19^, inflammatory bowel disease (IBD)^20^, and multiple sclerosis (MS)^21^. Nevertheless, it is unclear whether inhibition of the NF-κB pathway could serve as a therapeutic target in immune-mediated inflammatory disorders such as NMO. Our findings revealed that hsAQP-IgG immune-mediated responses lead to the release of pro-inflammatory factors in astrocytes, which is regulated by the NF-κB pathway. Therefore, we aimed to investigate whether inhibition of NF-κB activation could prevent astroglial activation and pro-inflammatory factor production in hsAQP4-IgG-treated astrocytes.

Recent studies have revealed that mesenchymal stem cells (MSCs)-derived exosomes and trophic factors can protect against tissue injury by regulating the activation of NF-κB and reducing the expression of pro-inflammatory cytokines (Fig. 1f)^22,23^. We cultured human umbilical cord-derived mesenchymal stem cells (hUC-MSCs) and conducted quality control tests, including assessment of morphology, phenotype, and differentiation (Supplementary Fig. 1e-h). We then collected the hUC-MSCs conditional media (MCM) and investigated whether MCM had a protective role in hsAQP4-IgG-treated mouse primary astrocytes (Fig. 1g). As expected, hsAQP4-IgG treatment led to AQP4 degradation and enhanced nuclear translocation of NF-κB-p65, which was significantly higher compared to the hsCtrl-IgG group as demonstrated by immunofluorescence analysis (Fig. 1h). Remarkably, administration of hUC-MSC conditional media (MCM) abolished AQP4 degradation and prevented NF-κB-P65 nuclear translocation (Fig. 1h). To further validate these findings, primary mouse astrocytes were treated with hsAQP4-IgG or hsCtrl-IgG, followed by treatment of hUC-MSC-MCM for 4 hours (Fig. 1g). Cytoplasmic and nuclear fractions were lysed and harvested simultaneously. The treated astrocyte lysates were then subject to immunoblotting to assay the protein levels of NF-κB-P65 and the control β-actin or histone H3 (Fig. 1i). Densitometric quantifications revealed that the MCM abrogated the role of hsAQP4-IgG-induced accumulation of active NF-κB-P65 in the cell nucleus (Fig. 1i). ELISA was then used to detect expression of the eleven representative NF-κB-targeted cytokines, which revealed significantly enhanced secretion of these cytokines in the hsAQP4-IgG treated group (Fig. 1i). However, MCM treatment significantly alleviated the inflammatory cytokine release in hsAQP4-IgG-treated astrocytes (Fig. 1j). This suggests that hUC-MSCs could block NF-κB activation and reduce release of inflammatory cytokines by secreting trophic factors.

### hUC-MSCs mitigate motor neuron loss and improve motor function in a systemic mouse model of NMO

**Fig. 2:**
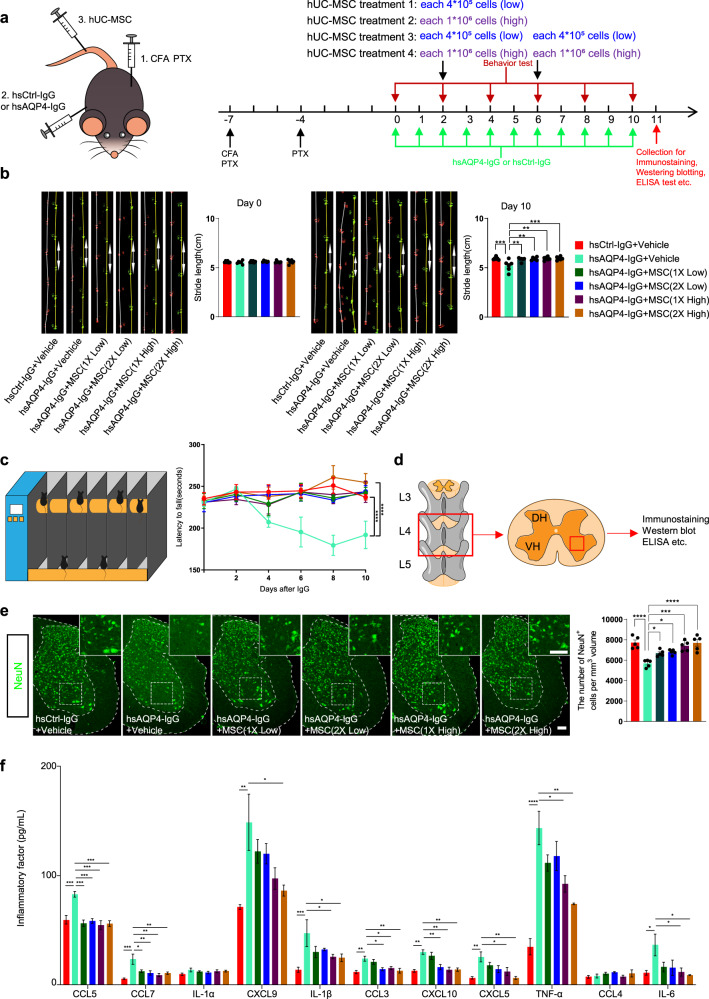
hUC-MSCs improves motor dysfunction in a systemic mouse model of NMO. **a** Schematic diagram and experimental procedures for assessing motor performance in NMO mouse models with hUC-MSCs treatment. (1) Subcutaneous injections of 50 μL of complete Freund’s adjuvant (CFA) containing 50 μg heat-killed H37Ra mycobacterium tuberculosis per site and 200 ng of pertussis toxin (PTX) were administered intraperitoneally (i.p.) twice to disrupt the blood–brain barrier (BBB) before IgG transfer. (2) From day 0 to 10, mice received daily I.P. injections of hsAQP4-IgG or hsCtrl-IgG. Each mouse received 4 mg of human IgG per day for 10 days (green row). (3) The NMO mouse models received hUC-MSCs treatment. Four groups of hUC-MSCs treatment were administered: after 2 days of IgG injection, the mice were treated with hUC-MSCs at the following doses: 4 × 10^5^ cells per mouse, one time tail vein injection (1x Low group); 1 × 10^6^ cells per mouse, one time tail vein injection (1x High group); 4 × 10^5^ cells per mouse, two times tail vein injection on the day 2 and day 6 (2x Low group); and 1 × 10^6^ cells per mouse, two times tail vein injection (2x High group). The standardized behavioral tests of the rotarod and gait test were performed on days 0, 2, 4, 6, 8, and 10. **b** Representative images and quantification of stride length on days 0 and 10. The data show that mice administered hsAQP4-IgG presented with reduced stride length on day 10, and hUC-MSCs treatment ameliorated the motor impairments. *n* = 6 animals per group. **c** Schematic diagram and quantification of the rotarod test. The data show that the hsAQP4-IgG-treated mice had an increased tendency to fall compared with hsCtrl-IgG-treated mice. hUC-MSCs treatment improved the motor impairment by extending the average latency time interval at which mice fell off. *n* = 6 animals per group. **d** Schematic diagram showing the cross-section of L4 spinal cord that were used for immunostaining, western blot, and ELISA analysis. **e** Representative confocal images and quantification of NeuN^+^ ventral horn motor neurons of the indicated groups. The data show that hUC-MSCs treatment ameliorated NeuN^+^ positive cells induced by hsAQP4-IgG. *n* = 5 animals per group. Scale bar, 100 µm. **f** Bar graph of the 11 overlapped NF-κB-targeted gene expression levels in L4 spinal cord lysates by ELISA analysis of the indicated groups. *n* = 3 animals per group. All data in the bar graphs of **b**, **c**, **e**, and **f** were presented as the mean ± SEM; the statistical evaluation of (**c**) was analyzed with two-way ANOVA with Tukey’s multiple comparisons test; for **b**, **e**, and **f**, the statistical significance was evaluated with one-way ANOVA and Tukey’s post hoc multiple comparisons. Non-significant comparisons are not identified. *p < 0.05, **p < 0.01, ***p < 0.001, ****p < 0.0001. CFA complete Freund’s adjuvant, PTX pertussis toxin.

Having established the inhibitory effect of hUC-MSCs on immune-induced NF-κB activation and pro-inflammatory factors released in vitro, we next sought to determine whether a similar effect could be observed in vivo. We optimized the passive immunization method to construct a mouse model of astrocytopathy similar to NMO, via passive transfer of the hsAQP4-IgG or hsCtrl-IgG to mice with BBB dysfunction^24^, which causes motor impairments (Fig. 2a and Supplementary Fig. 2a). We then examined the effects of hsAQP4-IgG on AQP4 and GFAP levels in spinal cord. Consistent with previous research reports, immunostaining revealed a marked decrease in AQP4 levels in the ventral horn of the spinal cord of mice treated with hsAQP4-IgG compared to hsCtrl-IgG treated mice (Supplementary Fig. 2b). However, the astrocyte cytoplasmic marker GFAP was significantly enhanced in the ventral horn after 6 days of hsAQP4-IgG injection (Supplementary Fig. 2c), consistent with sublytic histopathological observations in NMO patients^25^ and with neuropathology induced by CNS application of hsAQP4-IgG in rodents^26–28^. Additionally, the number of Iba1^+^ microglia (Supplementary Fig. 2d) was markedly increased in the ventral horn in mice injected with hsAQP4-IgG. Importantly, we found that the number of NeuN^+^ motor neuron (Supplementary Fig. 2e) in the ventral horn decreased significantly with hsAQP4-IgG treatment. This may be responsible for the initial behavioral impairment that follows hsAQP4-IgG binding to astrocytic AQP4. These findings suggest that hUC-MSCs may have therapeutic potential in NMO by inhibiting immune-induced NF-κB activation and reducing pro-inflammatory factor release, thereby protecting motor neurons from damage.

Subsequently, we investigated the effects of hUC-MSCs treatment on motor performance in mice using four different treatment strategies (Fig. 2a). On day 0, standardized behavioral tests including the rotarod and gait test were conducted, and no significant difference in motor performance was observed among the different treatment groups (Fig. 2b). However, on day 10, mice treated with hsAQP4-IgG exhibited reduced stride length (Fig. 2b), indicating impaired motor coordination. Additionally, hsAQP4-IgG-treated mice had a lower tendency of falling from the cylinder during the rotarod task compared to hsCtrl-IgG-treated mice (Fig. 2c). In contrast, administration of hUC-MSCs led to a robust improvement in behavioral performance in both low dose (single or twice injection) or high dose (single or twice injection) treatment groups (Fig. 2b, c). This strongly suggests that hUC-MSCs significantly alleviate motor impairments induced by hsAQP4-IgG treatment in mice, likely by preventing neuron dysfunction in the spinal cord.

We next aimed to investigate whether hUC-MSCs could alleviate motor neuron damage and attenuate the release of NF-κB-targets inflammatory factors. Results from in vitro experiments showed that hUC-MSCs prevented AQP4 degradation and reduced inflammatory cytokine release (Fig. 1g–j). To determine whether hUC-MSCs exerted the same effects in vivo, we used immunostaining analysis to evaluate NMO-like pathological changes in the ventral horn with hsAQP4-IgG injection (Fig. 2d). We found that hUC-MSCs markedly prevented the decrease in AQP4 (Supplementary Fig. 2f), and the number of NeuN^+^ motor neurons (Fig. 2e) in the ventral horn was significantly enhanced in all hUC-MSCs treatment groups. In contrast, the number of GFAP^+^ astrocytes (Supplementary Fig. 2g), Iba1^+^ microglia (Supplementary Fig. 2h), and TUNEL^+^ apoptotic cells (Supplementary Fig. 2i) was significantly reduced with hUC-MSCs treatment. Moreover, in line with our in vitro results, hUC-MSCs also reduced the activation of NF-κB (Supplementary Fig. 2j) and interrupted pro-inflammatory cytokine release (Fig. 2f) in NMO mice. Overall, transplantation of hUC-MSC improves motor function in NMO mice by preventing neuron loss and inflammatory factor release.

### MFGE8 secretion by hUC-MSCs inhibits NF-κB-targeting pro-inflammatory factors and ameliorates immune-induced motor impairments

After establishing the role of hUC-MSCs in NMO models in vitro and in vivo, we turned our attention to elucidating the mechanism by which core functional factors secreted from hUC-MSCs exerted important roles in modulating the recovery of motor function and release of pro-inflammatory factors. To achieve this, we used transcriptome analysis to quantify the expression levels of 355 known cytokines in hUC-MSCs from different generations (passage 4, 5, 6, 7, named 420F4-7) derived from umbilical cords of a single donor. Transcriptome profiling revealed that 106 secreted cytokines were highly expressed in hUC-MSCs of different generations (Supplementary Fig. 3a). To validate our data, we compared our results with a similar dataset of hUC-MSCs transcriptomes by Zhang et al., which was based on Illumina Hiseq 2500 analysis platform^29^. We observed an overlap in 94 out of 106 of these cytokine genes (Supplementary Fig. 3a) and the hierarchical clustering of heatmap based on these 94 overlapped cytokines was clearly demonstrated (Supplementary Fig. 3b). Subsequently, we identified 60 factors that exhibited the strongest signal with values greater than 100 (Supplementary Fig. 3c). Through detailed analysis of the role of each factor, we highlighted four factors that are likely to be involved in anti-inflammatory mechanisms: THBS1^30^, MFGE8^31,32^, MCP1(CCL2)^33^, and CST3^34^. In the in vitro hsAQP4-IgG-induced cells, we observed that only MFGE8 was able to restore the AQP4 or GFAP expression and inhibit activation of the NF-κB pathway (Supplementary Fig. 3d–f).

Examining the clinical significance of MFGE8 levels in NMO patients experiencing myelitis, we conducted a longitudinal analysis involving 45 patients with comprehensive disease records, alongside 44 age-matched healthy controls (Supplementary Table 2).

**Fig. 3:**
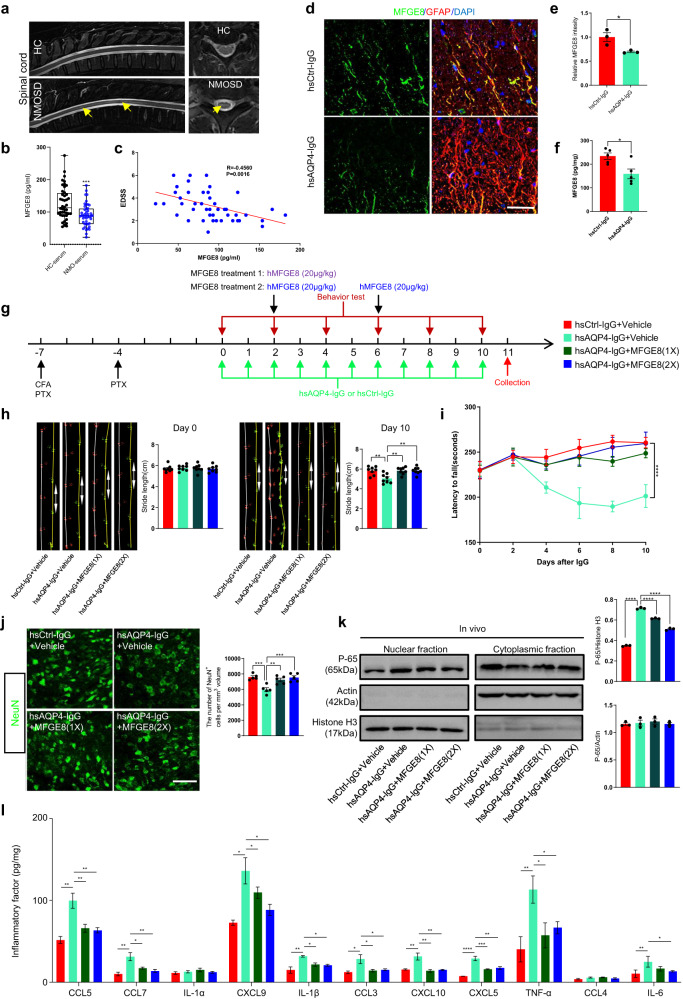
MFGE8 secreted from hUC-MSCs inhibits NF-κB_targeted pro-inflammatory factors release and ameliorates immune-induced locomotor impairments. **a** Representative MRI images of spinal cord in coronal and sagittal planes from healthy control (HC) subjects and NMO patients, with typical neuromyelitis lesions indicated by yellow arrows. **b** ELISA measurements of MFGE8 levels in serum samples from HC subjects and NMO patients (HC-serum, *n* = 44; NMO-serum, *n* = 45). **c** Correlation between serum MFGE8 levels and EDSS scores in NMO patients, showing a negative correlation (*n* = 45). **d** Representative confocal images of MFGE8 expression in astrocytes of L4 spinal cord tissues induced by hsAQP4-IgG. Scale bar, 100 µm. **e** Quantification of MFGE8 fluorescence intensity colocalized with GFAP signals, presented as relative levels with hsCtrl-IgG value set as 1.0 (*n* = 3 animals/group). **f** ELISA measurements of MFGE8 levels in unfixed L4 spinal cord tissue lysates from NMO mouse models (*n* = 5 animals/group). **g** Experimental timeline and procedures to assess MFGE8 role in NMO mouse model, similar to Fig. 2a design. **h** Quantification of stride length in indicated groups on day 0 and day 10, showing MFGE8 treatment ameliorates motor impairments in NMO mouse model (*n* = 8 animals/group). **i** Quantification of rotarod test, indicating MFGE8 treatment improves average latency time before mice fall off (*n* = 8 animals/group). **j** Representative confocal images and analysis of NeuN^+^ motor neurons in indicated groups, showing MFGE8 treatment prevents neuron loss in NMO mouse model (*n* = 8 animals/group). Scale bar, 100 µm. **k** Evaluation of MFGE8 effect on NF-κB signaling pathway activation in vivo: immunoblotting analysis of NF-κB-P65 and control β-actin or histone H3 in L4 spinal cord tissues collected after 10 days of IgG injection (*n* = 3 animals/group), revealing hsAQP4-IgG treatment-induced rapid accumulation of active NF-κB-P65 in cell nucleus, which is prevented by MFGE8 treatment. **l** Bar graph analysis revealing MFGE8 downregulates NF-κB target pro-inflammatory factor expression by ELISA tests (*n* = 3 animals/group). Data in bar graphs (**b**, **e**, **f**, **h**, **j**–**l**) presented as mean ± SEM; bar graphs (**c**) analyzed with Pearson’s coefficient; statistical evaluation of (**b**, **e**, **f**) performed with Student’s t-test for 2 conditions; evaluation of (**h**, **j**, **k**, **l**) performed with one-way ANOVA and Tukey’s post hoc multiple comparisons, for **l**, significance evaluated with two-way ANOVA and Tukey’s multiple comparisons test. Non-significant comparisons not identified. *p < 0.05, **p < 0.01, ***p < 0.001, ****p < 0.0001.

All patients were positive for autoantibodies against AQP4, and demographic data showed a predominance of young women (mean age: 43.20 ± 13.54 years; male/female = 4/41) (Supplementary Table 2). Strikingly, we observed that MFGE8 levels were significantly decreased in serum samples from NMO patients as compared to controls (Fig. 3b). Importantly we found a significant negative correlation between the EDSS score and MFGE8 levels in serum of NMO patients (Fig. 3c). Similarly, we also found the MFGE8 expression was reduced in NMO mouse model by immunostaining (Fig. 3d, e) and ELISA test of L4 spinal cord (Fig. 3f). These results indicate that the decreased expression of MFGE8 may contribute to motor dysfunction observed in NMO patients.

To assess our hypothesis that MFGE8 secretion by hUC-MSC contributes to recovery of motor function in NMO mice, we tested the effect of recombinant human MFGE8 protein (hMFGE8) on motor performance (Fig. 3g). We treated NMO mice with MFGE8 or vehicle and evaluated performance in gait and rotarod tests. MFGE8 administration robustly improved stride length in treated mice compared to controls (Fig. 3h). In parallel, MFGE8 administration ameliorated motor impairments by extending the average latency interval after which mice fell in the rotarod test (Fig. 3i). These findings indicate that MFGE8 markedly alleviated the motor impairments triggered by hsAQP4-IgG.

Next, we investigated the effect of hMFGE8 administration on neuronal damage. Supplementation of hMFGE8 lessened motor neuron loss (Fig. 3j) and AQP4^+^ fluorescent signaling (Supplementary Fig. 3g) in NMO mouse models, similar to the function of hUC-MSCs. Consistent with in vitro findings, hMFGE8 administration reduced the number of GFAP^+^ activated astrocytes (Supplementary Fig. 3h) and Iba1^+^ microglia (Supplementary Fig. 3i). Additionally, replenishment of hMFGE8 inhibited the activation of the NF-κB pathway (Fig. 3k) and reduced the release of inflammatory cytokines (Fig. 3l). These findings support the notion that MFGE8 plays a significant role in mediating hUC-MSCs-modulated recovery of motor deficits in NMO mouse model.

### Deletion of MFGE8 abolished the ability of hUC-MSCs to improve motor deficits

**Fig. 4:**
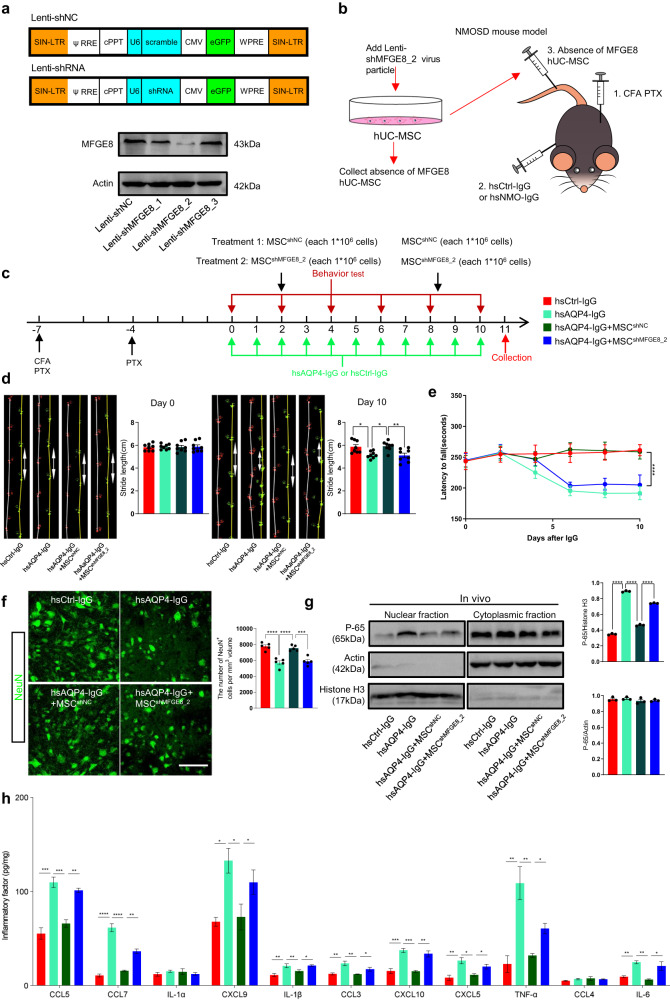
Deletion of MFGE8 abolished the capacity of hUC-MSCs to improve locomotor dysfunction. **a** Schematic diagram of lentiviral vector designs for transduction of shRNAs targeting MFGE8 for knockdown (shMFGE8_1, shMFGE8_2, shMFGE8_3) and scrambled non-targeting control shRNA (Lenti-shNC) using U6 promoter; eGFP coexpression driven by CMV promoter. Immunoblotting analyses (lower panel) evaluate knockdown efficiency of shRNAs against MFGE8 in cultured hUC-MSCs. **b** Schematic diagram illustrating experiment to test effects of hUC-MSCs-secreted MFGE8 on recovery of motor function in NMO mouse model. MSCs treated with Lenti-NC or Lenti-shMFGE8_2 for 72 hours; groups MSCs^shNC^ and -MSCs^shMFGE8_2^ collected and assessed for MFGE8 absence in relation to motor function improvement in NMO mouse model. **c** Experimental timeline and procedures for assessing roles of MSCs^shNC^ and MSCs^shMFGE8_2^ in NMO mouse model. **d** Representative images and quantification of stride length on day 0 and day 10, showing mice administered MSCs^shMFGE8_2^ lost ability to improve motor impairment (*n* = 8 animals/group). **e** Quantification of rotarod test, indicating hUC-MSCs^shMFGE8_2^ mice lost ability to improve motor impairment (*n* = 8 animals/group). **f** Representative confocal images (left panel) and quantification (right bar graphs) of NeuN^+^ ventral horn motor neurons in L4 spinal cord of indicated groups (*n* = 5 animals/group). Scale bar, 100 µm. **g** L4 spinal cord tissues collected from indicated groups at day 11, followed by cytoplasmic and nuclear fraction lysis and protein level analysis of NFκB-P65, β-actin, and histone H3 by immunoblotting. Densitometric quantifications (lower bar graphs) show hsAQP4-IgG treatment-induced rapid accumulation of active NF-κB-P65 in cell nucleus, with MSCs^shMFGE8_2^ losing ability of MSCs^shNC^ to prevent NF-κB-P65 entry into cell nucleus (*n* = 3 animals/group). **h** Bar graph of 11 overlapped NF-κB-P65 targeted gene expression levels in L4 spinal cord lysates by ELISA analysis for indicated groups (*n* = 3 animals/group). All data in bar graphs of **d**, **f**, **g**, and **h** presented as mean ± SEM; statistical evaluation of **d**, **f**, **g**, and **h** performed using one-way ANOVA and Tukey’s post hoc multiple comparisons; for **e**, statistical significance evaluated with two-way ANOVA and Tukey’s multiple comparisons test. Non-significant comparisons not identified. *p < 0.05, **p < 0.01, ***p < 0.001 ****p < 0.0001.

To confirm the pivotal role of MFGE8 in hUC-MSC-mediated motor function recovery in vivo, we utilized MFGE8-deficient hUC-MSCs to assess the contribution of MFGE8 to the therapeutic effects observed in NMO mouse models. Firstly, we designed three shRNA sequences targeting MFGE8, and transduced them individually through lentivirus (shMFGE8_1, shMFGE8_2, and shMFGE8_3) as well as non-targeting control shRNA (shNC) into cultured hUC-MSCs (Fig. 4a), with co-expression of EGFP to ensure adequate lentiviral transduction efficiency (of at least 95%). The efficiency of knockdown was confirmed by comparing MFGE8 protein levels in control conditions to those in shRNA-transduced cells, and shMFGE8_2 was selected for subsequent experiments (Fig. 4a). We then transplanted the MFGE8-deficient hUC-MSCs (MSCs^shMFGE8_2^) into NMO mouse models alongside the controls (MSCs^shNC^) (Fig. 4b, c). Subsequently, animals underwent gait and rotarod tests to evaluate locomotor function. In the gait test, the hsAQP4-IgG+MSCs^shNC^ group exhibited significantly improved stride length compared to the hsAQP4-IgG group (Fig. 4d). In contrast, the hsAQP4^-^IgG+MSCs^shMFGE8_2^ group did not exhibit significant improvement in gait test (hsAQP4-IgG+hUC-MSCs^shMFGE8_2^ group vs. hsAQP4-IgG+MSCs^shNC^ group) (Fig. 4d). Similarly, administration of MSCs^shNC^ improved motor impairments in the rotarod test by extending the average latency period at which mice fell from the cylinder (hsAQP4-IgG+MSCs^shNC^ group vs. hsAQP4-IgG group) (Fig. 4e). However, MFGE-deficient hUC-MSCs completely abolished the rescued effect on motor deficits (hsAQP4_IgG+ MSCs^shMFGE8_2^ group vs. hsAQP4_IgG+MSCs^shNC^group) (Fig. 4e).

Furthermore, immunostaining analysis revealed that, the MSCs^shNC^ group showed significant improvement in the relative fluorescence intensity of AQP4^+^ signals (Supplementary Fig. 4a) and the number of NeuN^+^ motor neurons (Fig. 4f) compared with hsAQP4-IgG group. Moreover, MSCs^shNC^ administration led to a decrease in the number of activated GFAP^+^ astrocytes (Supplementary Fig. 4b) and Iba1^+^ microglia (Supplementary Fig. 4c). However, the administration of MFGE8-deficient hUC-MSCs failed to inhibit AQP4^+^ signals (Supplementary Fig. 4a) and NeuN^+^ neurons loss (Fig. 4f), as well as the activation of astrocytes (Supplementary Fig. 4b) and microglia (Supplementary Fig. 4c) when compared to the MSCs^shNC^ group.

To assess the activitation of the NF-κB pathway and its associated pro-inflammatory factors. While the administration of exogenous MSCs^shNC^ significantly inhibited the NF-κB signaling pathway and limited the release of pro-inflammatory factors in vivo (hsAQP4-IgG+MSCs^shNC^ group vs. hsAQP4-IgG alone) (Fig. 4g, h), this inhibitory effect was absent in MFGE8-deficient hUC-MSCs (hsAQP4-IgG+MSCs^shMFGE8_2^ group vs. hsAQP4-IgG+ MSCs^shNC^ group) (Fig. 4g, h). Similarly, in vitro, absence of MFGE8-MCM was abolished the inhibition role of AQP4 signals loss and activation of NF-κB (Supplementary Fig. 4d, e). This suggests that MFGE8 plays a critical role in mediating the hUC-MSCs-modulated recovery of motor deficits in NMO mouse models.

### MFGE8-mediated inhibition of NF-κB signaling through integrin engagement reduces pro-inflammatory factor release and motor neuron loss

**Fig. 5:**
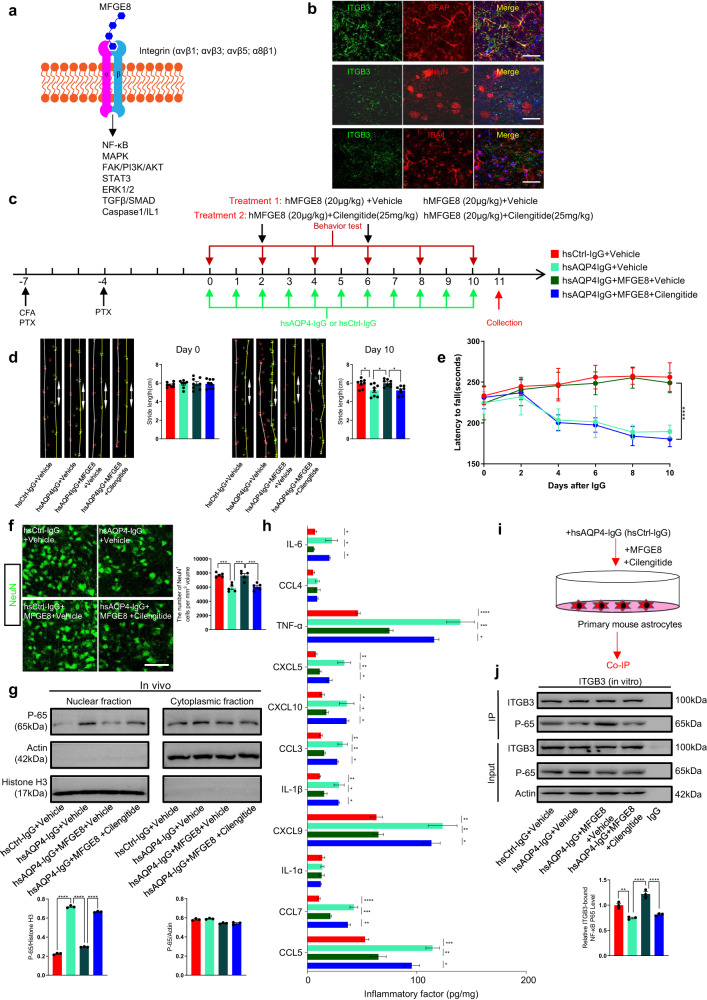
MFGE8 engages integrin αvβ3 to improve locomotor dysfunction and block NF-κB targeted pro-inflammatory factors release. **a** Summary of integrin receptor family and downstream signaling pathways activated by MFGE8-receptor binding, including NF-κB, MAPK, FAK/PI3K/AKT, STAT3, ERK1/2, TGFβ/SMAD, and Caspase1/IL1. **b** Expression of ITGB3 receptor on astrocytes but not neurons or microglia, as shown by immunostaining. Top row demonstrates colocalization of ITGB3 (green) and GFAP (red) fluorescence signals; little colocalization between ITGB3 (green) and neuronal marker NeuN (red) (middle panels) or microglia marker Iba1 (red) (bottom panels). Spinal cord slices from 8-week-old wild-type mice. Scale bars, 50 µm. **c** Experimental timeline and procedures to assess αvβ3-integrin inhibitor Cilengitide (25 mg/kg) role in NMO mouse model. **d** Representative images and quantification of stride length on days 0 and 10 in each group, showing Cilengitide inhibits MFGE8 treatment-associated motor impairment improvement (*n* = 8 animals/group). **e** Rotarod test quantification, showing MFGE8-treated (20 µg/kg) NMO model mice exhibit increased falling tendency compared to hsAQP4-IgG-treated mice, and Cilengitide inhibits MFGE8 action improving motor impairment (*n* = 8 animals/group). **f** Representative confocal images and quantification of NeuN^+^ ventral horn motor neurons for indicated groups (*n* = 5 animals/group). Scale bar, 100 µm. **g** After 10-day IgG injection, L4 spinal cord tissues collected on day 11 for indicated groups; cytoplasmic and nuclear fractions lysed and harvested simultaneously. Lysates subjected to immunoblotting for NF-κB-P65, β-actin, and histone H3 protein levels. Densitometric quantifications (right bar graphs) reveal Cilengitide prevents MFGE8 treatment-associated restraint of NF-κB-P65 nuclear entry (*n* = 3 animals/group). **h** Bar graph of 11 overlapping NF-κB-targeted gene expression levels in L4 spinal cord lysates, assessed by ELISA for indicated groups (*n* = 3 animals/group). **i** Primary mouse astrocytes treated with hsAQP4-IgG or hsCtrl-IgG, followed by MFGE8 or MFGE8 with Cilengitide treatment for 4 hours. Total proteins lysed and harvested simultaneously; treated astrocyte lysates subjected to co-IP to assay NF-κB-P65 and ITGB3 interaction. **j** Representative western blot images of NF-κB-P65 and ITGB3 interaction in indicated groups (left panels). Densitometric quantifications (right bar graph) reveal hsAQP4-IgG-treated primary mouse astrocytes exhibit diminished interaction between NF-κB-P65 and ITGB3 compared with hsCtrl-IgG-treated group. MFGE8 could enhance the effect that ITGB3 interacted with NF-κB-P65, and application of Cilengitide weakens the role of MFGE8 in vitro (*n* = 3 experiments/group). All data in bar graphs (**d**, **f**, **g**, **h**, **j**) presented as mean ± SEM; statistical evaluation of (**d**, **f**, **g**, **h**, **j**) performed using one-way ANOVA and Tukey’s post hoc multiple comparisons; for (e), significance evaluated using two-way ANOVA with Tukey’s multiple comparisons test. Non-significant comparisons not identified. *p < 0.05, **p < 0.01, ***p < 0.001, ****p < 0.0001.

We have demonstrated that MFGE8 can restore the expression of AQP4 and GFAP, inhibit the activation of the NF-κB pathway, and reduce inflammation both in vivo and in vitro (Fig. 3 and Supplementary Fig. 3). To elucidate the MFGE8 signaling mechanism responsible for motor function recovery in neuroinflammation, we aimed to identify the receptor and downstream pathway in astrocytes. The most well-characterized MFGE8 receptor is integrin αvβ1/3/5, and α8β1^35^ (Fig. 5a), but immunostaining analysis revealed that integrin β1 (ITGB1), integrin β5 (ITGB5), integrin α8 (ITGA8) were not expressed in astrocytes in the L4 spinal cord (Supplementary Fig. 5a). In contrast, integrin β3 (ITGB3) was found to be expressed in astrocytes, but not in Iba1^+^ microglia and NeuN^+^ neurons (Fig. 5b). With integrin αvβ3 identified as a potential MFGE8 receptor in astrocytes, we focused on the specific downstream pathway mediated by NF-κB (Fig. 5a).

We proceeded to investigate the involvement of integrin αvβ3 in mediating MFGE8 signaling using Cilengitide, a cyclicized pentapeptide containing arginine-glycine-aspartic acid, to block ανβ3 integrin activation^36,37^. Cilengitide administration (100 ng/mL) in vitro abolished the inhibitory effect of MFGE8 on the NF-κB pathway in astrocytes (Supplementary Fig. 5b, c). Additionally, Cilengitide blocked the anti-inflammatory effect of MFGE8 that by inducing GFAP expression and AQP4 degradation (Supplementary Fig. 5b). To validate these findings in vivo, we administered Cilengitide to the mice in the hsAQP4-IgG+MFGE8+Cilengitide group and sacrificed them at the indicated timepoints to prepare L4 spinal cord slices for confocal microscopy (Fig. 5c). Behavior tests found that Cilengitide could mark reverse the promoting effect of MFGE8 on locomotor function (Fig. 5d, e).

Immunostaining analysis further revealed that Cilengitide treatment abolished the beneficial effects of MFGE8 on recovery of motor neuron injury, as indicated by the relative fluorescence intensity of AQP4 (Supplementary Fig. 5d), and the number of NeuN^+^ motor neurons (Fig. 5f) compared to the hsAQP4-IgG+MFGE8+Vehicle group. Furthermore, Cilengitide administration also abolished the inhibitory effects of MFGE8 on astrocyte and microglia activation in the indicated groups, as demonstrated by the number of activated GFAP^+^ astrocytes (Supplementary Fig. 5e) and Iba1^+^ microglia (Supplementary Fig. 5f). These findings suggest that integrin αvβ3 plays a crucial role in mediating MFGE8 signaling, which ultimately leads to the recovery of motor deficits in neuroinflammation.

To explore how MFGE8 regulates the NF-κB pathway and associated pro-inflammatory factors. Exogenous administration of MFGE8 significantly inhibited the activation of NF-κB signaling (Fig. 5g) and the release of NF-κB-targeted pro-inflammatory factors (Fig. 5h) in vivo as compared to the hsAQP4-IgG+PBS group. However, this inhibitory effect was blocked by Cilengitide (Fig. 5g, h, hsAQP4-IgG+MFGE8 +Vehicle group vs. hsAQP4-IgG+MFGE8+Cilengitide group).

To explore the functional role of integrin αvβ3 in the regulation of the NF-κB pathway, we hypothesized that MFGE8 might enhance the interaction between ITGB3 and NF-κB-P65, thereby disrupting the translocation of NF-κB-P65 into the nucleus. to test this hypothesis, we first validated the interaction between ITGB3 and NF-κB-P65 in mouse primary astrocytes by co-immunoprecipitation (co-IP) assay (Supplementary Fig. 5g). We found that the interaction of NF-κB-P65 and ITGB3 was diminished in primary mouse astrocytes treated with hsAQP4-IgG (Fig. 5i, j). However, MFGE8 could enhance the binding of NF-κB-P65 and ITGB3, while weakened the role of MFGE8 through application of Cilengitide (Fig. 5j). Similar to in vitro, the effect of MFGE8 and ITGB3 in NF-κB-P65 could be observed in vivo (Supplementary Fig. 5h). These findings provide strong support for a mechanistic model in which MFGE8 acts as a suppressor of NF-κB activation through engagement of the integrin αvβ3 receptor, particularly under conditions of astrocyte activation in the context of neuroinflammation.

## Discussion

Neuroinflammation is a significant target of treatment across various CNS diseases, including Alzheimer’s disease^38^, glioblastoma^39^, and autoimmune disorders like multiple sclerosis^40^ and NMO^41^. Understanding the molecular mechanisms underlying inflammatory neurotoxicity after astroglial activation is crucial for developing next-generation anti-neuroinflammatory treatments. Treatments incorporating MSCs have been investigated for various immune-mediated conditions, including graft versus host disease, Crohn’s disease, and multiple sclerosis, due to their stem cell multipotency and robust anti-inflammatory and regenerative properties^10^. A pilot trial has demonstrated that MSC infusion is safe and can reduce relapse frequency and mitigate neurological disability, with recovery of neural structures in the optic nerve and spinal cord in patients with NMO^42^.

In this study, we have shown that the NF-κB pathway and NF-κB-targeted pro-inflammatory factors are the primary effectors of immune-mediated astroglial activation in compromised spinal cord function during neuroinflammation. Using primary astrocytes and mouse models of NMO, we demonstrated that MSC-derived MFGE8 improves the motor deficits in NMO mouse models by inhibiting motor neuron loss and reducing the release of inflammatory factors targeted by NF-κB. Specifically, MFGE8 interacts with the ITGB3 receptor on astrocytes and triggers a downstream signaling pathway mediated by NF-κB to decrease the translocation of NF-κB-P65 to the cell nuclei and the expression of inflammatory factors, further reducing motor neuron injury. Supplementation of MFGE8 or pharmacological inhibition of the NF-κB pathways could rescue locomotor function. In summary, our findings provide new insight into the role of MFGE8 in the spinal cord and offer a potential therapeutic approach for immune-mediated neuroinflammatory disorders.

The NF-κB pathway has long been recognized as a master regulator of autoimmunity, playing a pivotal role in both inflammation and immune tolerance^43,44^. While transient activation of NF-κB is a normal part of immune response, prolonged activation of this signaling pathway in target tissues has been associated with the pathogenesis of many autoimmune disorders^45^. Therapeutic targeting of the NF-κB signaling pathway is thus a promising strategy for controlling the progression from normal immunity to autoimmunity^44,46,47^. Recent research has revealed that cytokines related to the NF-κB pathway are significantly elevated in individuals with NMO during the acute and remission phases^48^, suggesting that this pathway may represent a potential therapeutic target for the disease. Other studies have demonstrated that hsAQP4-IgG, which is associated with the development of NMO, stimulates the activation of the canonical NF-κB signaling pathway in astrocytes^16^. To verify these findings, we utilized an astroglial culture system to examine the cellular and molecular events induced by hsAQP4-IgG stimulation.

Our findings also showed that hsAQP4-IgG led to the engagement of a highly inflammatory and reactive astrocyte transcriptional program, which included the upregulation of numerous pro-inflammatory factors encoded by the NF-κB pathway, such as IL-6, TNFα, and IL-1β. We further confirmed the involvement of the NF-κB pathway by observing an increase in nuclear translocation of the NF-κB transcription factor NF-κB-P65 in astrocytes following hsAQP4-IgG stimulation, as demonstrated by immunostaining and immunoblotting analysis. Accumulating evidence suggests that MSCs exert their anti-inflammatory effects through downregulation of NF-κB pathway activation in immune diseases. However, whether hUC-MSCs have an impact on pathological locomotor dysfunction in NMO remains to be determined.

In this study, we comprehensively evaluated the effects of MSCs on NMO cell and mouse models. Our findings revealed that MSC treatment improved motor deficits by reducing NF-κB-targeted inflammatory factors through the downregulation of NF-κB signaling. Moreover, we confirmed that MFGE8 was the core functional factor from MSCs that plays an essential role in the hUC-MSCs-mediated recovery of locomotor deficits, at least in part through the inhibition of the ITGB3/NF-κB signaling pathway. The present study provides valuable insights into the therapeutic potential of MSCs in NMO and the underlying molecular mechanisms involved. Our findings demonstrate that hUC-MSCs can significantly improve motor deficits in NMO mouse models by downregulating the NF-κB pathway and reducing the production of NF-κB-targeted pro-inflammatory factors. Moreover, we identified MFGE8 as a core functional factor derived from MSCs that plays a crucial role in the modulation of the ITGB3/NF-κB signaling pathway and subsequent recovery of locomotor deficits. MFGE8 is known to be involved in various physiological functions and has been found to have therapeutic potential in various CNS diseases, including neurodegenerative diseases^49,50^ and cerebral ischemic injury^51^. Our study further confirms the importance of MFGE8 in NMO and suggests that supplementation with human recombinant MFGE8 could be an effective therapeutic strategy. Schwann cell-derived exomes containing MFGE8 have been found to improve the recovery of motor function after spinal cord injury^52^, and our study provides further evidence of the beneficial effects of MFGE8 in NMO.

We also identified ITGB3 as the receptor for MFGE8 in astrocytes, and our findings suggest that MFGE8-mediated anti-inflammatory effects are generated through NF-κB inhibition by modulating αvβ3 integrin signaling. These results are consistent with previous studies that have reported enhanced NF-κB-targeted pro-inflammatory factors in NMO patients^53–55^ and primary astrocytes treated with hsAQP4-IgG^16,56–61^.

In the NMO groups, both astrocytes and microglia are activated, and the interaction between astrocytes and microglia is considered to play an important role in the development of NMO^28^. However, the interaction between astrocytes and microglia is still not fully elucidated. Previous reports have suggested that exogenous supplementation of MFGE8 could exert anti-inflammatory effects^52,62,63^, our study also found that MFGE8 treatment could inhibit the activation of microglia and inflammatory factors release. However, further investigation is required to study MFGE8-mediated signaling pathway in microglia in NMO models.

Finally, our study provides strong evidence for the therapeutic potential of hUC-MSCs and MFGE8 in NMO and highlights the importance of the NF-κB pathway in the pathogenesis of the disease. These findings raise the possibility that MFGE8 or NF-κB inhibitors may be used as alternative therapies for NMO. Further studies are needed to validate these findings in human clinical trials and to investigate the potential side effects of long-term treatment.

## Methods

### Study design

We used hUC-MSCs to examine whether MSC-derived functional core factors play a positive role to the recovery of locomotor deficits in the NMO mouse model. Five experimental studies were designed: (1) We found that hUC-MSCs conditional medium inhibits NF-κB-targeted pro-inflammatory cytokines release in mouse primary astrocytes by immunostaining, Western blotting, and ELISA assays; (2) We used behavioral tests of the rotarod and gait, Western blotting, ELISA, immunostaining to evaluate the function of hUC-MSCs in improving locomotor dysfunction and decreasing inflammation in NMO mouse model; (3) We identified hUC-MSCs secreted MFGE8 could restore NMO related lesions and motor deficits, and inhibit activation of NF-κB pathway by transcriptome analysis, behavioral tests, Western blotting, ELISA and immunostaining in vitro and in vivo; (4) We determined the therapeutic effect of recombinant MFGE8 though engaging with ITGB3 receptor to ameliorate motor dysfunction by using ITGB3 inhibitor, behavioral tests, Western blotting, ELISA, immunostaining, and co-IP assay. Animals were randomly assigned to different experimental groups and researchers were blinded to the genotype of the animals until the experiments were completed. No statistical method was used to predetermine the sample size for each experiment. No data or animals were excluded from the analysis. In vitro experiments were repeated more than three times, with the replication described in each figure legend.

### hUC-MSCs preparation and conditioned medium collection

Written informed consent was obtained from the participants before umbilical cord collection. The processes including isolation, culture, identification, quality control, and storage of hUC-MSCs were strictly conformed to standard operating procedures established by Guangzhou SALIAI Stem cell Science and Technology Co., Ltd. The preparation of hUC-MSC was conducted as follows: In a biological safety cabinet, umbilical cord in sterile physiological saline was transferred to a sterile stainless-steel disc containing DMEM/F12 basal medium. The umbilical cord was then cut into pieces with tissue scissors. Next, 0.25% type IV collagenase was added for digestion and samples were subsequently shaken at 37 °C for 2 hours. Precipitated cells were centrifuged at 300×*g* for 5 min and washed with DMEM/F12 three times, followed by seeding of the cell suspension in a 150 mm cell culture dish and culture in complete medium (DMEM/F12 supplemented with 10% FBS, 1% nonessential amino acids, and 2 mM L-glutamine). The primary hUC-MSCs were harvested when cell confluence reached 80% and then expanded by regular subculture.

The positive markers (CD73, CD105, CD90) and negative markers (CD11b, CD19, CD34, CD45, HLA-DR) of passage 2 (P2) and P5 hUC-MSCs were detected by flow cytometry (Beckman, DxFLEX). The cell surface markers were identified according to the criteria of positive marker expression rate ≥ 95% and negative marker ≤ 2%, proposed by the mesenchymal and tissue stem cell committee of the International Society for Cell Therapy (ISCT). The following fluorescently labelled antibodies were used: PE anti-human CD73 (1:100, BioLegend, 344004), APC anti-human CD90 (1:100, BioLegend, 328114), APC anti-human CD34 (1:100, BioLegend, 343608), Alexa Fluor® 488 anti-human CD45 (1:100, BioLegend, 304017), PE anti-human CD11b (1:100, BioLegend, 301306), PE/Cyanine7 anti-human CD19 (1:100, BioLegend, 302216), and PE/Cyanine7 anti-human HLA-DR (1:100, BioLegend, 307616) and Alexa Fluor® 488 anti-human CD105 (1:100, Abcam, ab187575).

The osteogenic, adipogenic and chondrogenic differentiation potential of hUC-MSCs were detected using osteogenic, adipogenic, and chondrogenic induction media respectively. The osteogenic and adipogenic differentiation media were homemade, while the chondrogenic differentiation media was purchased from GIBCO (Cat. No. A1007101) and used following the manufacturer’s instructions. Successful inductions of adipogenesis, osteogenesis, and chondrogenesis were confirmed by staining with Oil Red O, Alizarin Red, and Safranine O.

hUC-MSCs conditioned medium was prepared as follows: hUC-MSCs were cultured at p5 in 150 mm cell culture dishes until they reached 80% confluence. Cells were washed twice with phosphate-buffered saline (PBS) to remove the complete medium, then cultured in DMEM/F12 basal medium for 48 h. The conditioned medium was collected and centrifuged at 300 g for 5 min to remove cellular debris.

### hUC-MSC Bulk-seq analysis

RNA samples from cultured hUC-MSCs were sequenced using a standard Illumina protocol (Novogene Co., Ltd.). Reads were mapped to the human genome (GRCh38) using STAR (v2.7.6a). Gene counts were estimated by HTSeq (v0.6.1) and gene TPM (Transcripts Per Kilobase Million) was calculated with gencode annotation (v35) in R (v3.5.2). Public hUC-MSC and our hUC-MSCs expression data from healthy donors was downloaded from GSE165811 and GSE246608 (https://www.ncbi.nlm.nih.gov, GEO).

Known cytokines were collected from Abcam website (www.abcam.com). High expression genes identified in own data and public data were filtered by mean of log1p TPM expression larger than 2. Intersected genes between the two datasets were identified and known cytokines were represented in an expression heatmap (pheatmap, v1.0.12) and Venn diagram (VennDiagram, v1.6.20) in R.

### Animals

Mice were kept in the animal facility of the Guangdong Laboratory Animals Monitoring Institute for 12 hours of back light/dark cycle. 8 to 12-week-old adult healthy female mice were used in experiments. The mice could obtain food and water in the cage ad libitum. All mice in the study were backcrossed to C57BL/6 background for at least six generations. The animals were randomly assigned to the experimental group. Researchers were blinded to the different groups. All procedures and feeding were carried out in accordance with the National Institutes of Health Guide for the Care and Use of Laboratory Animals approved by the Animal Care Committee and the Ethics committee of the Third Affiliated Hospital of Sun Yat-sen University. Mice were anesthetized by isoflurane before injection. To collect tissue samples, mice were anesthetized by isoflurane and euthanized.

### Primary astrocyte culturing and treatment

Primary astrocytes were isolated from mice, purified, and used for primary culture as described previously^17,18^. Briefly, mixed cortical glial cells were harvested from neonatal mouse pups at postnatal day 0 (P0) after removal of the meninges and maintained in DMEM/F12 containing 10% FBS, 100 µM nonessential amino acids, 2 mM l-glutamine, 1 mM sodium pyruvate, and 1% penicillin–streptomycin for 14 days at 37°C and 5% CO_2_ and medium was replaced every 3 days. Primary astrocytes were plated onto poly-L-lysine-coated glass coverslips in 24-well plates at a density of 8 × 10^3^ cells per well. The cultures were treated with controls or biological reagents at the indicated final concentration for the described experimental durations: PBS (vehicle), LPS (100 ng/mL, Beyotime), hsAQP4-IgG (100 ng/mL), hsCtrl-IgG (100 ng/mL), MFGE8 (100 ng/mL, Signalway Antibody), THBS1 (100 ng/mL, Signalway Antibody), CST3 (100 ng/mL, Signalway Antibody), and CCL2 (100 ng/mL, Sino Biological). Astrocyte cultures were incubated with 20% MSCs^shNC^ conditioned medium (or MSCs^shMFGE8_2^ conditioned medium) or other mentioned reagents for 4 hours at 37 °C and were harvested by fixation with 4% paraformaldehyde (PFA) for immunostaining, or by lysis buffers for Western blotting, RNA-seq, qPCR assays, and ELISA.

### Human IgG purification

Total human IgG was purified from the plasma of seropositive NMO patients who received plasma exchange and healthy volunteers using protein A beads (GE Healthcare, 71149800-EG). The beads were eluted with 0.1 mol/L glycine-HCl (pH 2.5), and the eluent was then concentrated using Amicon Ultra15 centrifugal filter units (100 kD, Millipore). The concentrated IgG preparation was sterilely filtered through a 0.22 μm filter, the IgG concentration was measured by BCA protein assay kit (KeyGen BioTECH), and working aliquots were stored at −80 °C. The pooled IgG was referred to as hsAQP4-IgG and hsCtrl-IgG.

### NMO mouse models

A systemic mouse model of NMO with evolving motor injury was established as described previously^24,64^ with some modifications. Mice were anesthetized and received subcutaneous injections of complete Freund’s adjuvant (CFA) (Sigma-Aldrich) containing heat-killed H37Ra Mycobacterium tuberculosis (BD-DIFCO) at four sites (50 μg in 50 μL of CFA per site) on the hind flank 7 days before IgG transfer. Furthermore, these animals received intraperitoneal injections of pertussis toxin (PTX, 200 ng in 0.2 mL of ddH_2_O, Enzo Life Science) 7 and 4 days prior to the IgG transfer to disrupt the blood–brain barrier (BBB). Between day 0 and day 10, systemic transfer of hsAQP4-IgG or hsCtrl-IgG (4.0 mg IgG in 0.2 mL PBS daily) was performed. The vehicle-treated mice were given 200 µL PBS by tail intravenous administration. NMO mice were treated with hUC-MSCs at a dose of 1 × 10^6^ cells or 4 × 10^5^ cells by tail intravenous administration. MFGE8 treatment (400 ng in 200 µL PBS) was administered to each mouse through the tail intravenous route.

### Behavioral test

Behavioral testing was performed on days 0, 2, 4, 6, 8, and 10 following IgG systemic transfer. The rotarod task test was performed using a 6-lane apparatus, which required mice to balance and walk on a rotating cylinder, starting at 10 rounds per minute and uniformly accelerating over a 5-min period to 40 rounds per minute. Each mouse was tested three times in 15-min intervals and the time of latency to fall was calculated as the mean of three times. Gait analysis was performed using the Catwalk-assisted gait analysis system (XR-FP101, SHXINRUAN), which consisted of mice walking freely across a narrow strip. The length of the hindlimb stride was calculated as the mean of five sequential steps.

### Immunofluorescence staining

Mice were euthanized by intraperitoneal injection of 2% pentobarbital sodium and flushed with PBS. Animals underwent trans-cardiac perfusion with 4% PFA. Spinal cords were fixed in 4% PFA at 4 °C overnight, and then balanced in 30% sucrose until submerged. The mouse L4 spinal cords were cut into 40-μm-thick sections coronally using a microtome and placed in a 96-well plate. The slices were kept in cryoprotectant solutions (glycerol, ethylene glycol, and 0.1 M phosphate buffer, at pH 7.4, 1:1:2 by volume) at 4 °C. For immunofluorescence staining, floating sections were blocked with 300 μL/10 mL goat serum and 250 μL /10 mL 10% Triton X-100 for 1 hour at room temperature and then sequentially incubated with primary antibodies overnight at 4 °C. Samples were then washed with PBS three times and incubated with a secondary antibody at room temperature for 1 hour. Nuclei were then stained with 4’, 6-dimethyl-2’- phenylindole dihydrochloride (DAPI, Sigma Aldrich, #b2261). Slides were visualized under a laser confocal microscope. Each set of immunofluorescence experiments was repeated at least 3 times. The following primary antibodies were used: rabbit anti-AQP4 (1:500, Introvigen, PA5-53234), mouse anti-GFAP (1:1000, CST, 3670 S), rabbit anti-Iba1 (1:1000, Wako Chemicals, 019-19741), mouse anti-NeuN (1:500, Abcam, ab104224), rabbit anti-MFGE8 (1:1000, Introvigen, PA5-109955), rabbit anti-integrin-β1 (1:200, Proteintech, 12594-1-AP), rabbit anti-integrin-β3 (1:200, Proteintech, 18309-1-AP), rabbit anti-integrin-β5 (1:200, Proteintech, 28543-1-AP), rabbit anti-integrin-α8 (1:200, Santa Cruz Biotechnology, sc-365798), and mouse anti-NF-κB-P65 (1:200, CST,6956). The following fluorescent secondary antibodies were used: goat anti-mouse 488 (1:1000, Invitrogen), goat anti-mouse 568 (1:1000, Invitrogen), goat anti-mouse 647 (1:1000, Invitrogen), goat anti-rabbit 488 (1:1000, Invitrogen), goat anti-rabbit 568 (1:1000, Invitrogen), goat anti-rabbit 647 (1:1000, Invitrogen), and goat anti-chicken 647 (1:1000, Invitrogen). Confocal single plane images and z-stacks were taken with a laser confocal microscope (Leica, TCS SP8) equipped with four laser lines (405, 488, 568, and 647 nm) and ×63, ×40, and ×20 objective lenses.

### Detection of apoptotic cells

Terminal deoxynucleotidyl transferase dUTP nick-end labeling (TUNEL) staining was performed using the TUNEL apoptosis detection kit (Yeasen, 40308ES) according to the manufacturer’s instructions. In brief, section of spinal cords was incubated with equilibration buffers for 10 min at room temperature, then were incubated with TUNEL reacting mixture for 1 hour at 37 °C.

### Cytoplasmic and nuclear extraction

Cytoplasmic and nuclear extracts from primary astrocytes or mouse L4 spinal cord tissue were separated using Nuclear and Cytoplasmic Protein Extraction Kit (Solarbio R0050) according to the manufacturer’s protocol. Place 20–30 mg fresh spinal cord tissue or primary astrocyte cells (2 × 10^6^) in a 1.5 ml tube with 500 μL PBS. Gently grind the tissue with the pestle provided using back and forth twisting force for about 1 min (40-60 times). Centrifuge at 500 × *g* for 2–3 min to collect cells, and remove the supernatant and leave the precipitation. 200 μL of plasma protein extraction reagent was added for every 20 μL cells or tissue precipitation. The cell or tissue precipitation need completely dispersed into a single cell suspension following incubate the tube on ice for 10 min. Centrifuge at 14,000 × *g* for 10 min and transfer supernatant to a fresh 1.5 mL tube (cytoplasmic protein). The pellet contains isolated nuclei and resuspend the pellet in 50 μL nuclear protein extraction reagent. Centrifuge the tube at 14,000 × *g* for 10 min. Transfer the supernatant to a fresh tube (nuclear protein). The nuclear and cytoplasmic proteins is suitable for immunoblotting analysis as described below.

### Western blotting and densitometric analysis

Nuclear protein, cytoplasmic protein, and total protein samples were lysed from primary astrocytes or spinal cord tissue using radioimmunoprecipitation assay (RIPA) lysis buffer. The proteins were separated on 8–12% sodium dodecyl sulfate-polyacrylamide gel by electrophoresis, and then transferred to methanol-activated polyvinylidene fluoride membranes. The membranes were blocked in 5% skim milk for 1 h at room temperature and incubated with primary antibodies overnight at 4 °C. The membranes were then washed with TBST three times and incubated with horseradish peroxidase (HRP) secondary antibodies for 1 hour. The primary antibodies were used: mouse anti-NF-κB-P65 (1:1000, CST, 6956), mouse anti-β-actin (1:1000, HuABio, EM21002), mouse anti-histone H3 (1:1000, HuABio, M1309-1), rabbit anti-MFGE8 (1:1000, Introvigen, PA5-109955). Protein bands were visualized with Millipore ECL Plus reagent and imaged on a Tanon 5500 Imaging Analysis System. The intensity of the bands was quantified using ImageJ software. All blots and gels originated from a single experiment and were treated concurrently. Complete scans can be found in supplemental figs. S6 and S7.

### Co-immunoprecipitation assay

For immunoprecipitation assay (IP), total protein was extracted from mouse spinal cord tissue or 2 × 15 cm dishes of primary astrocytes with full confluence, according to the manufacturer’s protocol (Beyotime Biotechnology P2179S). The total protein lysates were immunoprecipitated with specific antibodies targeting against ITGB3 (1:50, Proteintech, 18309-1-AP), NF-κB-P65 (1:50, CST, 6956), or IgG negative control at 4 °C overnight on a rotator. Samples were then incubated with 50 μL Protein A/G Magnetic Beads (pre-cleaned with PBST) on a rotator for 2 h at 4 °C. Subsequently, magnetic beads were washed 4 times with PBST buffer, and immunoprecipitation complexes were separated from the beads for Western blot experiments.

### RT-qPCR

mRNA from primary astrocytes was extracted with the miRNeasy kit (Qiagen). Then, mRNA was quantified and checked for purity using a Nanodrop spectrophotometer (ThermoFisher Scientific). The cDNA was converted from 1 µg mRNA using the SureScript First-Strand cDNA Synthesis Kit (Genecopoeia Company). RT‒qPCR was performed using BlazeTaq SYBR Green qPCR Mix (Genecopoeia Company) with Applied Biosystems (Thermo Fisher Scientific). Fold changes were calculated as (2 – ΔΔCT) with GAPDH used as the endogenous control. The primers used are listed in Supplementary Table 3.

### Clinical data collection

Patients were recruited at the department of neurology from The Third Affiliated Hospital of Sun Yat-sen University. NMO patients were diagnosed according to the 2015 International Panel for NMO Diagnosis (IPND) criteria. Serum of NMO patients and healthy controls were obtained with written informed consent from all participants. All patients included in this study were recorded in demographics and medical history, including pathogenetic characteristics, disease duration, years of education and EDSS scores. Blood samples were collected within seven days of symptom onset during clinical remission, or during a stable stage when the patient was relapse-free for at least 3 months and all patients underwent thorough neurological examination with assessment of the EDSS by a trained EDSS rater. This study was approved by the Ethics Committee of the Third Affiliated Hospital of Sun Yat-sen University, in strict accordance with the Declaration of Helsinki.

### MRI acquisition and EDSS scores

MRI data was obtained using a 3.0 T magnetic resonance system (Philips Medical System Ingenia scanner) with dStream head coil. Structural images of the whole brain were scanned using 3D fast spoiled gradient-echo sequence. FLAIR data were scanned using TR = 7000 ms, Flip Angle 90, TE = 125 ms, acquisition matrix = 272 × 176, and slice thickness of 6 mm.

The disability severity of NMO was evaluated by the EDSS score. The symptom severity was rated as mild disability (EDSS score from 0 to 3.5) or moderate/severe disability (EDSS score from 4.0 to 9.5). An EDSS score of 4.0 is considered: limited walking without assistance, and an EDSS score of 6.0 is considered: unilateral assistant assistance walking. The EDSS scores of 4.0 and 6.0 are regarded as key milestones in disability and irreversible disability.

### Inhibitor administration

In vitro, astrocytes cultures were treated with Cilengitide (10 μM, Selleck) for 4 hours at 37 °C. Cilengitide was administered by intraperitoneal injection at the dosage of 25 mg/kg on days 2 and days 6.

### Production of lentivirus particles

The lentiviruses (lenti-shNC and lenti-shMFGE8) were routinely produced as described previously^65^, through the transfection of human embryonic kidney (HEK293T) cells (ATCC, CRL-11268) with the desired vectors. Briefly, HEK293T cells were maintained at 37 °C in a CO_2_ cell incubator (Thermo Fisher 371) in Dulbecco’s Modification of Eagle’s Medium (DMEM) (319-005-CL, MULTICELL) containing 10% fetal bovine serum, 1% penicillin–streptomycin, and 1% glutamine. Cells were passaged every 3 days with 0.25% trypsin EDTA. For the production of lentivirus particles, the designed and cloned lentiviral vectors were co-transfected with helper plasmids psPAX and pMD2G into HEK293T cells by using polyethyleneimine (PEI, PR40001, Proteintech Group). The culture medium was changed within 4 hours. The medium containing lentivirus was collected at 2 to 4 days post-transfection, filtered, and concentrated by ultracentrifugation (Beckman SW32 Ti). The viruses were washed once with Dulbecco’s phosphate-buffered saline (DPBS) and then resuspended in 200 μL DPBS. Each tube was labeled and stored at −80 °C.

### Pro-inflammatory factors analysis by ELISA

Samples of mouse spinal cord, primary astrocytes, serum were obtained from experimental NMO model animals or NMO patients and health controls. Blood samples were spun for 15 min at 8000 rpm at 4 °C to yield serum. Levels of inflammatory factor were measured with commercial ELISA kits based on the provided directions:

CCL5: (MEIMIAN, MM-0903M1); CCL7: (MEIMIAN, MM-0084M1);

IL-1α: (MEIMIAN, MM-0168M1); CXCL9: (MEIMIAN, MM-44928M1);

IL-1β: (MEIMIAN, MM-0040M1); CCL3: (MEIMIAN, MM-0154M1);

CXCL10: (MEIMIAN, MM-45188M1); CXCL5: (MEIMIAN, MM-0499M1);

TNF-α: (MEIMIAN, MM-0132M1); CCL4: (MEIMIAN, MM-0153M1);

IL-6:(MEIMIAN, MM-0163M1). Levels of MFGE8 in spinal cord from mice were measured with commercial ELISA kits (MEIMIAN, MM-0694M1). Levels of MFGE8 in serum from NMO patients and health controls were measured with commercial ELISA kits (MEIMIAN, MM-1704H1). The absorbance of each standard and sample was measured at 450 nm. A standard concentration gradient was used as a standard curve.

### Statistical analysis

All data in bar graphs and summary plots were shown as means ± standard error (SEM) of at least three independent biological replicates in all figures. No statistical method was used to estimate sample size, as pre-specified effect sizes were not assumed. The scale bars and samples sizes were all described in the figure legends. Normality of the data distribution was assessed by a Shapiro-Wilk normality test (*p* < 0.05 indicating a non-normal distribution). We used Student’s t test for pair-wise comparisons, one-way or two-way ANOVA followed by Tukey’s post-hoc test for 3 groups or more for normally distributed data. The correlation between EDSS score and the MFGE8 expression of NMO patients was performed using the Pearson correlation coefficient. In addition, descriptive statistics were used to examine the demographic characteristics, medical history information and EDSS testing of HCs or patients. Significance was reported as *p < 0.05, **p < 0.01, ***p < 0.001, or ****p < 0.0001. All statistical analyses were performed in GraphPad Prism 9 (GraphPad Software).

### Supplementary information

Supplementart information

reporting-summary

## Data Availability

Our expression date for hUC-MSCs during this study have been deposited in the GEO database (GSE246608). Public expression date for hUC-MSCs is available in the GEO database (GSE165811). All other data collected and analyzed during the current study are available from the corresponding author upon reasonable request.

## Code availability

All datasets were generated and processed with publicly available software. The software, software version, and websites were described in the “Methods” section.
