# Supplementary material for: hUC-MSCs-derived MFGE8 ameliorates locomotor dysfunction via inhibition of ITGB3/ NF-κB signaling in an NMO mouse model
Source: NPJ Regen Med. 2024 Jan 20;9:4. doi: 10.1038/s41536-024-00349-z (PMC10798960; doi:10.1038/s41536-024-00349-z)
Supplement: Supplementary file 1 — Supplementart information [file 41536_2024_349_MOESM1_ESM.pdf]

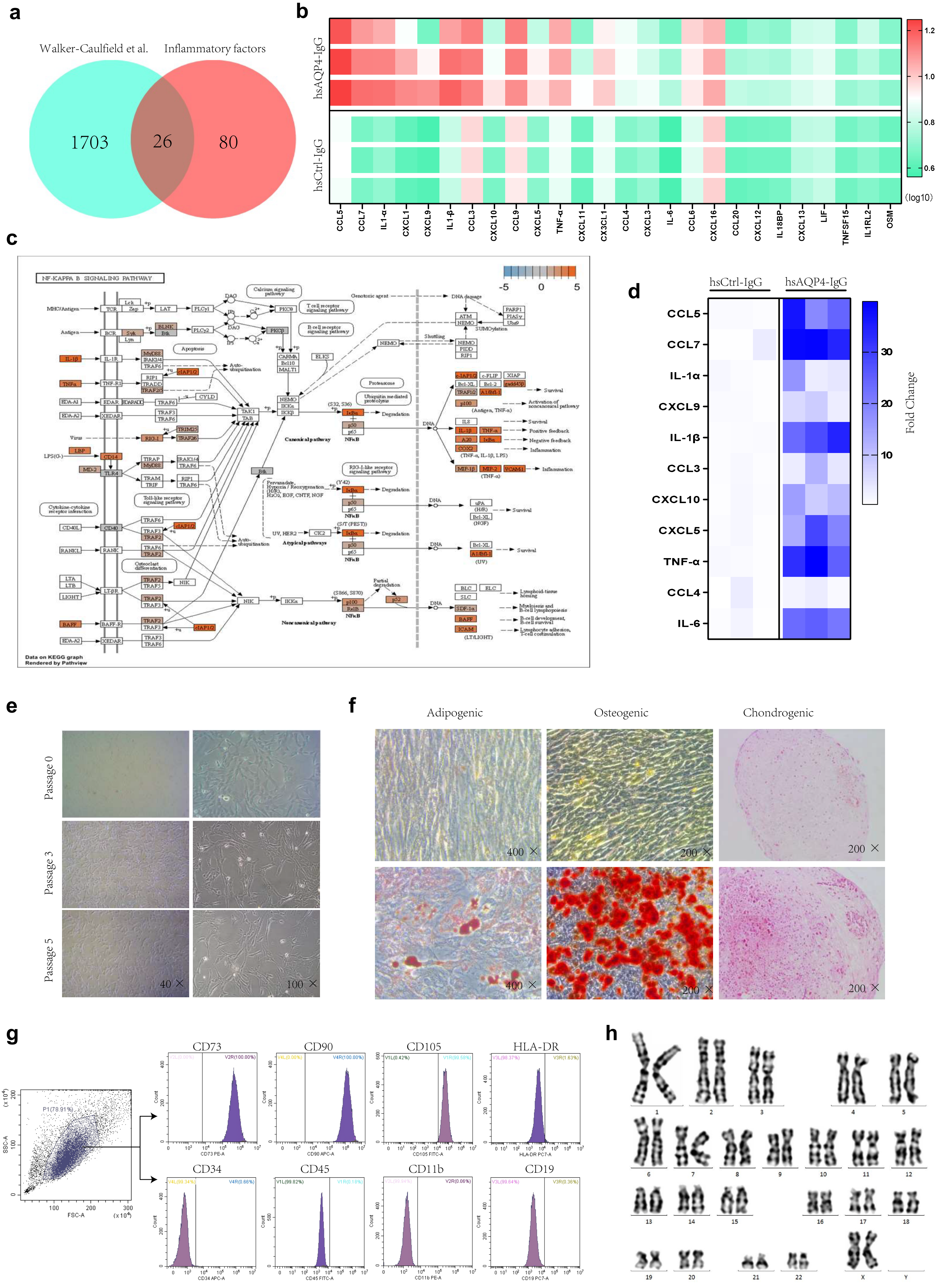
a
Walker-Caulfield et al.
Inflammatory factors
1703
26
80
b
hsAQP4-IgG
hsCtrl-IgG
1.2
1.0
0.8
0.6
(log10)
CCL5
CCL7
IL1-α
CXCL1
CXCL9
IL1-β
CCL3
CXCL10
CCL9
CXCL5
TNF-α
CXCL11
CX3CL1
CCL4
CXCL3
IL-6
CCL6
CXCL16
CCL20
CXCL12
IL18BP
CXCL13
LIF
TNFSF15
IL1RL2
OSM
c
NF-KAPPA B SIGNALING PATHWAY
-5
0
5
Canonical pathway
Atypical pathways
Noncanonical pathway
Data on KEGG graph
Rendered by Pathview
d
hsCtrl-IgG
hsAQP4-IgG
CCL5
CCL7
IL-1α
CXCL9
IL-1β
CCL3
CXCL10
CXCL5
TNF-α
CCL4
IL-6
30
20
10
Fold Change
e
Passage 0
Passage 3
Passage 5
40 ×
100 ×
f
Adipogenic
Osteogenic
Chondrogenic
400 ×
200 ×
200 ×
400 ×
200 ×
200 ×
g
CD73
CD90
CD105
HLA-DR
CD34
CD45
CD11b
CD19
P1(78.91%)
SSC-A
FSC-A
V2R(100.00%)
V4R(100.00%)
V1R(99.58%)
V3L(98.37%)
V3R(1.63%)
V4L(99.34%)
V4R(0.66%)
V1L(99.82%)
V1R(0.18%)
V2R(0.06%)
V3L(99.64%)
V3R(0.36%)
CD73 PE-A
CD90 APC-A
CD105 FITC-A
HLA-DR PC7-A
CD34 APC-A
CD45 FITC-A
CD11b PE-A
CD19 PC7-A
h
1
2
3
4
5
6
7
8
9
10
11
12
13
14
15
16
17
18
19
20
21
22
X
Y

Supplementary Figure 1. Additional data of transcriptional analyses on mouse astrocytes treated with hsAQP4-IgG and characterization of hUC-MSCs

(a) Venn diagram showing the overlap of differentially expressed genes (DEGs) identified by transcriptomic analysis of mouse astrocytes stimulated with human anti-AQP4 antibodies (hsAQP4-IgG) or control human IgG (hsCtrl-IgG). The diagram shows that 26 out of 106 pro-inflammatory cytokines were upregulated by hsAQP4-IgG. The green portion represents the results of Walker-Caulfield et al.,[16] and the red portion represents the pro-inflammatory database.

(b) Gene fold changes in the overlapped 26 pro-inflammatory cytokines expression levels, plotted on a log10 scale in comparison to untreated samples to unmask the effect of control IgG treatment. Zero-fold change is shown in green, and upregulation >+1.5-fold is shown in red.

(c) NF-κB canonical pathway identified via pathway visualization of the 1729 upregulated DEGs by extracting the KEGG with the data integration tool of Pathview.

(d) RT-qPCR assays confirmed that the 11 overlapped NF-κB target pro-inflammatory genes were upregulated by hsAQP4-IgG in mouse primary astrocytes. The data of qPCR were plotted as the heatmap of gene fold change. White color indicates no change, and blue indicates >40-fold change. n=3 independent experiments.

(e) Representative morphology of hUC-MSCs. Magnification: ×40 and ×100.

(f) Representative images of hUC-MSCs differentiated into adipocytes, osteocytes, and chondrocytes are shown as indicated. Successful inductions of adipogenesis, osteogenesis, and chondrogenesis were confirmed by staining with Oil Red O (left panels), Alizarin Red (middle panels), and Safranine O (right panels). Magnification: ×200 and ×100.

(g) Gating strategy for flow cytometry analysis of hUC-MSCs. FSC/SSC: forward scatter/sideward scatter.Flow cytometric analysis showed that hUC-MSCs were positive for mesenchymal lineage markers (CD73, CD90, and CD105), negative for hematopoietic and endothelial markers (CD34, CD45, CD11b, CD19), and negative for HLA-DR.

(h) Representative karyotype analysis of hUC-MSCs (Passage 15, a normal male karyotype).

Abbreviations: DEGs, differentially expressed genes; NF-κB, nuclear factor-kappa B; qPCR, quantitative polymerase chain reaction; hUC-MSCs, human umbilical cord mesenchymal stem cells; KEGG, Kyoto Encyclopedia of Genes and Genomes.

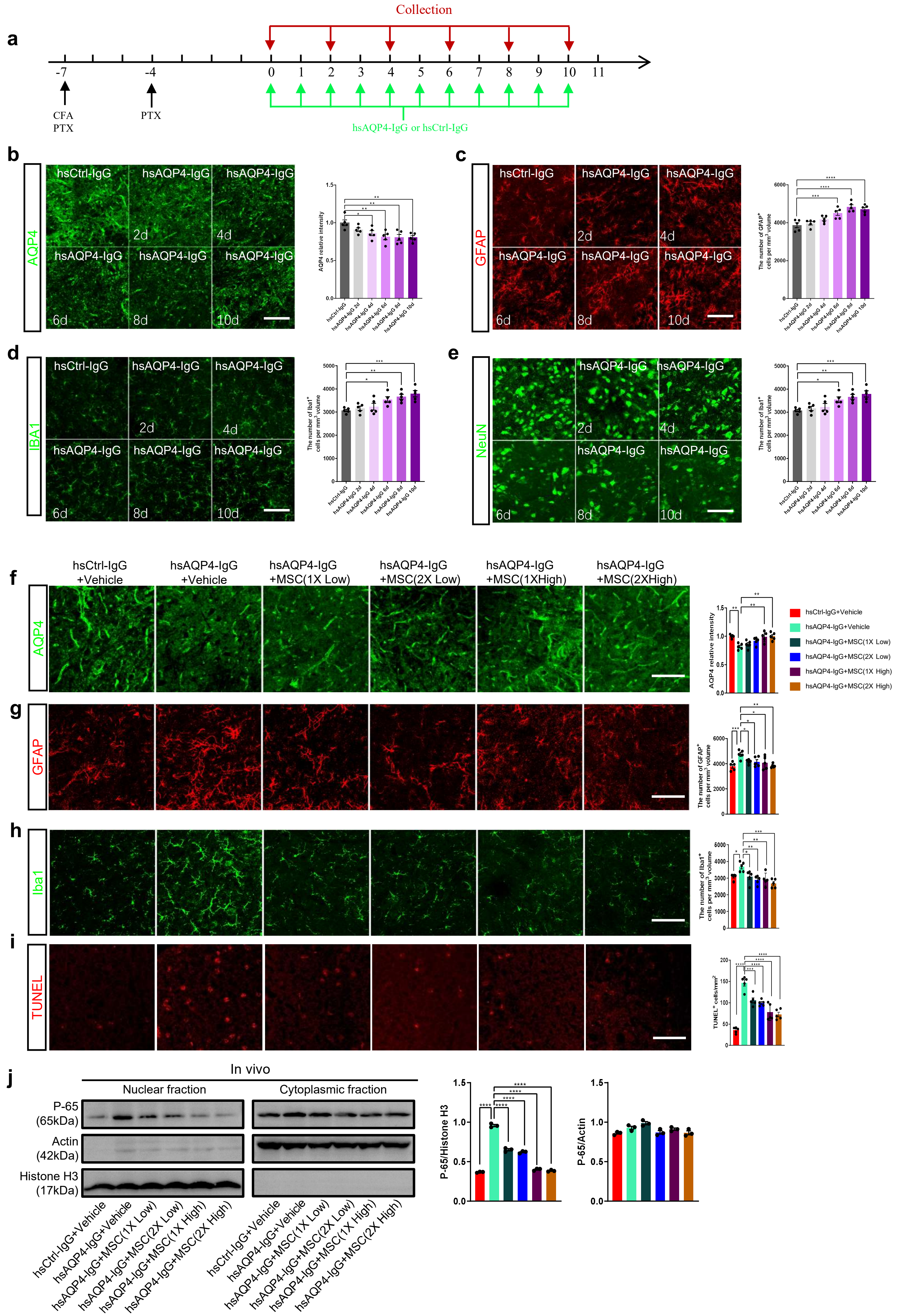

a
Collection
-7
-4
0
1
2
3
4
5
6
7
8
9
10
11
CFA
PTX
PTX
hsAQP4-IgG or hsCtrl-IgG
b
AQP4
hsCtrl-IgG
hsAQP4-IgG
2d
4d
6d
8d
10d
AQP4 relative intensity
c
GFAP
The number of GFAP+ cells per mm3 volume
d
IBA1
The number of Iba1+ cells per mm3 volume
e
NeuN
f
hsCtrl-IgG +Vehicle
hsAQP4-IgG +Vehicle
hsAQP4-IgG +MSC(1X Low)
hsAQP4-IgG +MSC(2X Low)
hsAQP4-IgG +MSC(1XHigh)
hsAQP4-IgG +MSC(2XHigh)
hsCtrl-IgG+Vehicle
hsAQP4-IgG+Vehicle
hsAQP4-IgG+MSC(1X Low)
hsAQP4-IgG+MSC(2X Low)
hsAQP4-IgG+MSC(1X High)
hsAQP4-IgG+MSC(2X High)
g
h
Iba1
i
TUNEL
TUNEL+ cells/mm2
j
In vivo
Nuclear fraction
Cytoplasmic fraction
P-65 (65kDa)
Actin (42kDa)
Histone H3 (17kDa)
P-65/Histone H3
P-65/Actin

Supplementary Figure 2. Characterization of NMO mouse model at different time point and the effect of hUC-MSCs on glia cell response

(a) Timeline and experimental procedures for establishing NMO mouse models, similar to Fig. 2A design. Mice were collected on day 0, 2, 4, 6, 8, and 10 for further analysis.

(b) AQP4 relative intensity in indicated groups, showing a gradual decrease in hsAQP4-IgG-treated group compared to hsCtrl-IgG group (n=5 animals/group).

(c) $GFAP^+$ astrocytes in L4 spinal cord ventral horn, indicating gradual reactivation in hsAQP4-IgG-treated group compared to hsCtrl-IgG group (n=5 animals/group).

(d) $Iba1^+$ microglia in L4 spinal cord ventral horn, indicating gradual reactivation in hsAQP4-IgG-treated group compared to hsCtrl-IgG group (n=5 animals/group).

(e) $NeuN^+$ motor neurons in L4 spinal cord ventral horn, showing a gradual decrease in hsAQP4-IgG-treated group compared to hsCtrl-IgG group (n=5 animals/group).

(f) AQP4 positive fluorescence signals in L4 spinal cord ventral horn, showing a robust decrease in hsAQP4-IgG-treated group compared to hsCtrl-IgG group, but hUC-MSCs treatment prevents signal loss (n=5 animals/group).

(g) $GFAP^+$ astrocytes in L4 spinal cord ventral horn, indicating gradual reactivation in hsAQP4-IgG-treated group compared to hsCtrl-IgG group, but hUC-MSCs treatment inhibits reactivation (n=5 animals/group).

(h) $Iba1^+$ microglia positive fluorescence signals in L4 spinal cord ventral horn, showing a robust increase in hsAQP4-IgG-treated group compared to hsCtrl-IgG group, but hUC-MSCs treatment inhibits reactivation (n=5 animals/group).

(i) $TUNEL^+$ positive cells in L4 spinal cord ventral horn, indicating a robust increase in hsAQP4-IgG-treated group compared to hsCtrl-IgG group, but hUC-MSCs treatment inhibits apoptotic cell reactivation (n=5 animals/group).

(j) Immunoblotting analysis of NF-κB-P65 and control β-actin or histone H3 in L4 spinal cord tissues collected after 10 days of IgG injection (n=3 animals/group), revealing hsAQP4-IgG treatment-induced rapid accumulation of active NF-κB-P65 in the cell nucleus, which is prevented by different doses of hUC-MSCs treatment.

Data in bar graphs (b-j) are presented as mean ± SEM; statistical evaluation was performed with one-way ANOVA and Tukey's post hoc multiple comparisons. Non-significant comparisons not identified. *$p<0.05$, **$p<0.01$, ***$p<0.001$, ****$p<0.0001$.

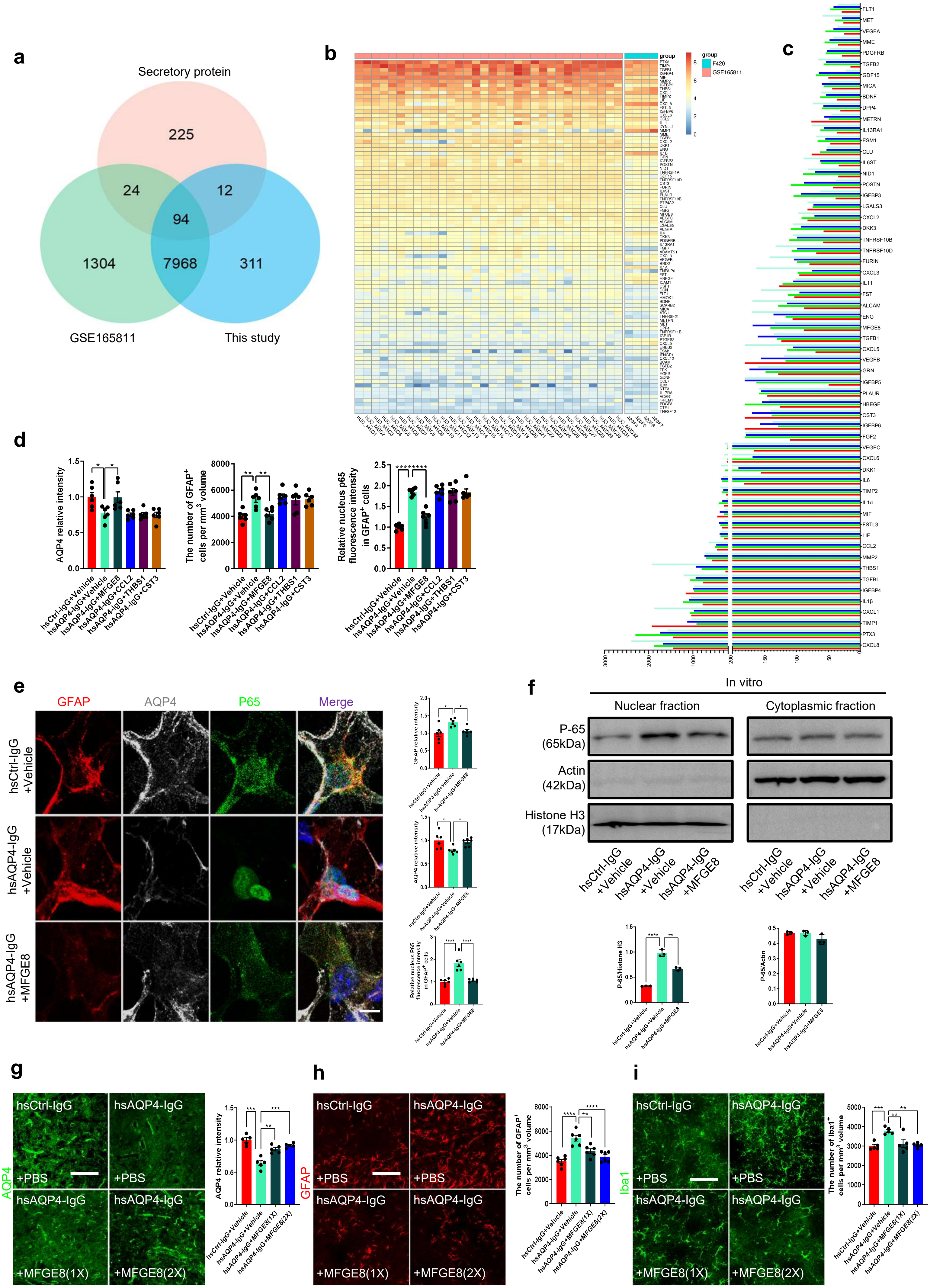

a
Secretory protein
225
24
12
94
1304
7968
311
GSE165811
This study
b
group
F420
GSE165811
c
d
AQP4 relative intensity
The number of GFAP+ cells per mm3 volume
Relative nucleus p65 fluorescence intensity in GFAP+ cells
hsCtrl-IgG+Vehicle
hsAQP4-IgG+Vehicle
hsAQP4-IgG+MFGE8
hsAQP4-IgG+CCL2
hsAQP4-IgG+THBS1
hsAQP4-IgG+CST3
e
GFAP
AQP4
P65
Merge
hsCtrl-IgG +Vehicle
hsAQP4-IgG +Vehicle
hsAQP4-IgG +MFGE8
f
In vitro
Nuclear fraction
Cytoplasmic fraction
P-65 (65kDa)
Actin (42kDa)
Histone H3 (17kDa)
hsCtrl-IgG +Vehicle
hsAQP4-IgG +Vehicle
hsAQP4-IgG +MFGE8
P-65/Histone H3
P-65/Actin
g
hsCtrl-IgG +PBS
hsAQP4-IgG +PBS
hsAQP4-IgG +MFGE8(1X)
hsAQP4-IgG +MFGE8(2X)
AQP4
AQP4 relative intensity
h
GFAP
The number of GFAP+ cells per mm3 volume
i
Iba1
The number of Iba1+ cells per mm3 volume

Supplementary Figure 3. Transcriptional analyses of hUC-MSC identifies MFGE8 preventing astrocytopathy and NF-κB signaling activation *in vitro*

(a) Venn diagram showing relative expression of hUC-MSCs and secretory proteins identified by transcriptomic study, with 94 overlapping genes from GSE165811 database and secretory proteins database. GSE165811 results in green, secretory protein database in red, and this study genes database in blue.

(b) Heatmap of high expression of 94 cytokine genes shared in two MSCs RNAseq datasets, including 32 samples from GSE165811 and 4 samples from this study. Public hUC-MSCs expression data from healthy donors downloaded from GSE165811 (https://www.ncbi.nlm.nih.gov, GEO); hUC-MSCs of fourth to sixth generations (420F4-420F7) from this study calculated as TPM. High expression abundant genes filtered by mean log1p TPM expression >2. Zero-fold change in blue and log1p TPM expression >10 in red.

(c) Relative expression levels of top 60 secretory cytokines from 94 overlapping genes in hUC-MSCs (420F4-420F7).

(d) Quantification of AQP4 relative intensity (left bar graph), $GFAP^+$ astrocytes (middle bar graph), and $Iba1^+$ microglia (right bar graph) positive fluorescence signals in spinal cord L4 ventral horn of indicated groups, showing MFGE8 treatment most effective in ameliorating AQP4 signal loss and inhibiting microglia and astrocytes reactivation compared to CCL2, THBS1, and CST3 (n=6 animals/group).

(e) Primary cultures of wildtype mouse astrocytes treated with hsAQP4-IgG (100ng/mL) and hsCtrl-IgG (100ng/mL), followed by MFGE8 treatment (100ng/ml) for 4 hours. Representative confocal images (left panels) of GFAP (red), AQP4 (gray), and NF-κB-P65 (green) in indicated groups. Quantification of fluorescence signals (right bar graphs) shows MFGE8 abrogates hsAQP4-IgG-induced active NF-κB-P65 accumulation in cell nucleus (n=6 experiments/group). Scale bar, 20 μm.

(f) Primary mouse astrocytes treated with hsAQP4-IgG or hsCtrl-IgG, followed by MFGE8 treatment for 4 hours. Cytoplasmic and nuclear fractions lysed and harvested simultaneously. Astrocyte lysates subjected to immunoblotting for NF-κB-P65 and control β-actin or histone H3. Densitometric quantifications (lower bar graphs) reveal MFGE8 abrogates hsAQP4-IgG-induced active NF-κB-P65 accumulation in cell nucleus (n=3 experiments/group).

(g) Representative confocal images and quantification of AQP4 positive fluorescence signals in L4 spinal cord ventral horn of indicated groups, showing AQP4 signal decrease in hsAQP4-IgG-treated group compared to hsCtrl-IgG group, but MFGE8 treatment prevents signal loss (n=5 animals/group). Scale bar, 100 μm.

(h) Representative confocal images and quantification of $GFAP^+$ astrocytes in L4 spinal cord ventral horn of indicated groups, showing increased $GFAP^+$ astrocytes in hsAQP4-IgG-treated group compared to hsCtrl-IgG group, but MFGE8 treatment inhibits astrocyte reactivation (n=5 animals/group). Scale bar, 100 μm.

(i) Representative confocal images and quantification of $Iba1^+$ microglia positive fluorescence signals in L4 spinal cord ventral horn of indicated groups, showing increased $Iba1^+$ microglia in hsAQP4-IgG-treated group compared to hsCtrl-IgG group, but MFGE8 treatment inhibits microglia reactivation (n=5 animals/group). Scale bar, 100 μm.

All data in bar graphs of (d-i) are presented as mean ± SEM; statistical evaluation of (d-i) performed using one-way ANOVA and Tukey's post hoc multiple comparisons. Non-significant comparisons not identified. *p<0.05, **p<0.01, ***p<0.001, ****p<0.0001.

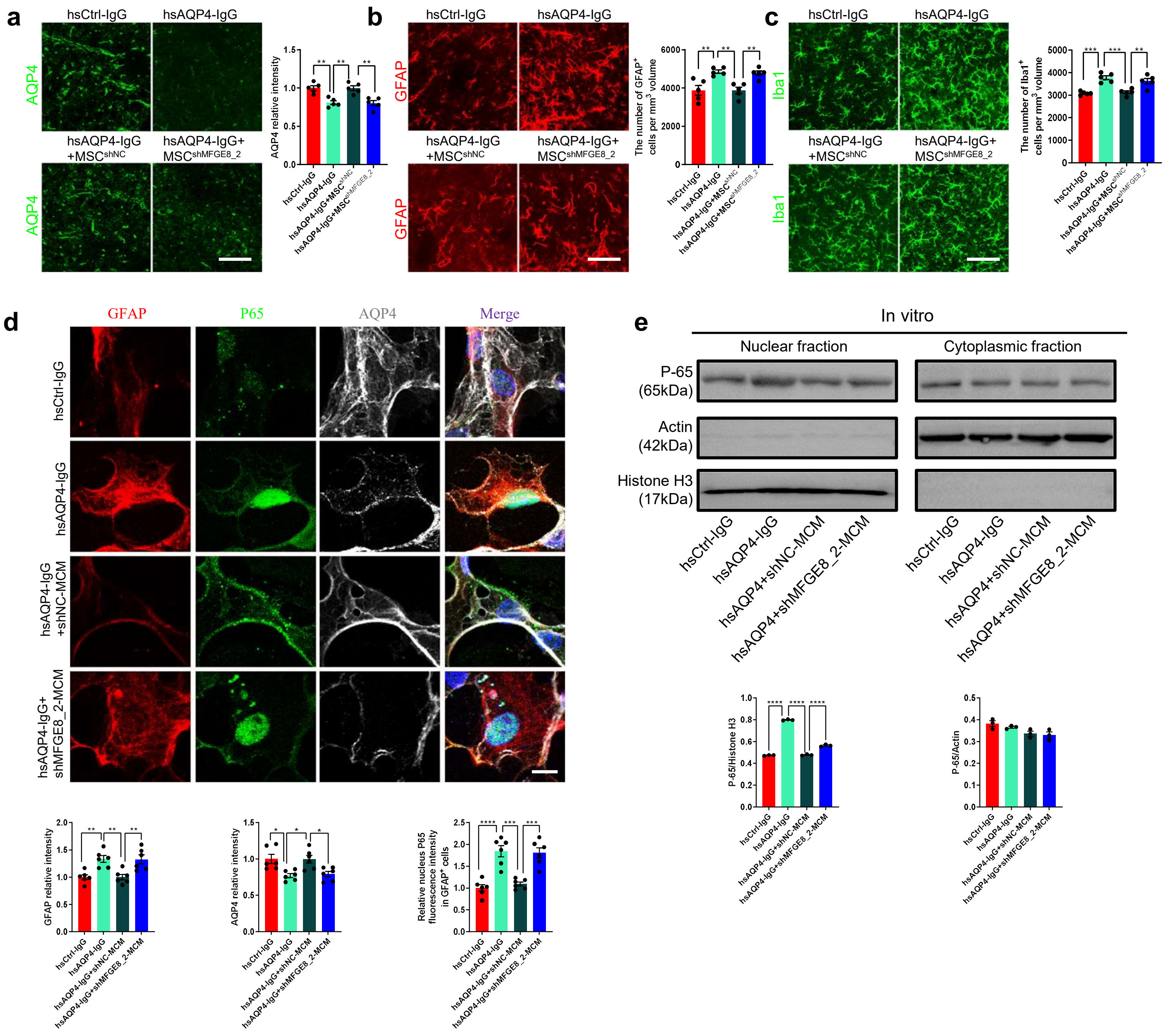
a
hsCtrl-IgG
hsAQP4-IgG
hsAQP4-IgG +MSCshNC
hsAQP4-IgG+ MSCshMFGE8_2
AQP4
AQP4 relative intensity
b
GFAP
The number of GFAP+ cells per mm3 volume
c
Iba1
The number of Iba1+ cells per mm3 volume
d
GFAP
P65
AQP4
Merge
hsCtrl-IgG
hsAQP4-IgG
hsAQP4-IgG +shNC-MCM
hsAQP4-IgG+ shMFGE8_2-MCM
GFAP relative intensity
AQP4 relative intensity
Relative nucleus P65 fluorescence intensity in GFAP+ cells
hsAQP4-IgG+shNC-MCM
hsAQP4-IgG+shMFGE8_2-MCM
e
In vitro
Nuclear fraction
Cytoplasmic fraction
P-65 (65kDa)
Actin (42kDa)
Histone H3 (17kDa)
hsCtrl-IgG
hsAQP4-IgG
hsAQP4+shNC-MCM
hsAQP4+shMFGE8_2-MCM
P-65/Histone H3
P-65/Actin

Supplementary Figure 4. hUC-MSC conditional medium absence of MFGE8 loss the function of inhibiting astrocytopathy and NF-κB signaling activation *in vitro*

(a) Representative confocal images and quantification of AQP4 positive fluorescence signals in L4 spinal cord ventral horn for indicated groups, showing $MSCs^{shNC}$ treatment prevents hsAQP4-IgG-induced AQP4 fluorescence signal absences and $MSCs^{shMFGE8\_2}$ group loss the inhibitory effect (n=5 animals/group). Scale bar, 100 μm.

(b) Representative confocal images and quantification of $GFAP^{+}$ astrocytes in L4 spinal cord ventral horn for indicated groups, showing $MSCs^{shNC}$ treatment inhibits astrocyte reactivation and $MSCs^{shMFGE8\_2}$ increases $GFAP^{+}$ astrocyte number compared to $MSCs^{shNC}$ treatment group (n=5 animals/group). Scale bar, 100 μm.

(c) Representative confocal images and quantification of $Iba1^{+}$ microglia positive fluorescence signals in L4 spinal cord ventral horn for indicated groups, showing increased $Iba1^{+}$ microglia number in $MSCs^{shMFGE8\_2}$ group compared to $MSCs^{shNC}$ treatment group (n=5 animals/group). Scale bar, 100 μm.

(d) $MSCs^{shNC}$ and $MSCs^{shMFGE8\_2}$ conditioned media harvested simultaneously; primary wildtype mouse astrocytes treated with hsAQP4-IgG (100 ng/mL) or hsCtrl-IgG (100 ng/mL), followed by MCM or $MSCs^{shMFGE8\_2}$-MCM treatment for 4 hours. Primary mouse astrocytes used for immunostaining, with representative confocal images (upper panels) showing GFAP (red), AQP4 (gray), and NF-κB-P65 (green) for indicated groups. Quantification of fluorescence signals (lower bar graphs) demonstrates $MSCs^{shMFGE8\_2}$-MCM treatment-induced active NF-κB-P65 nuclear accumulation, diminishing $MSCs^{shNC}$-MCM effect (n=6 experiments/group). Scale bar, 20 μm.

(e) Primary mouse astrocytes treated with hsAQP4-IgG or hsCtrl-IgG, followed by $MSCs^{shNC}$-MCM or $MSCs^{shMFGE8\_2}$-MCM treatment for 4 hours. Cytoplasmic and nuclear fractions lysed, and treated astrocyte lysates subjected to immunoblotting for NF-κB-P65, β-actin, and histone H3 protein level analysis. Densitometric quantifications (lower panels) reveal shMFGE8_2-MCM diminishes $MSCs^{shNC}$-MCM-induced active NF-κB-P65 nuclear accumulation (n=3 experiments/group).

All data in bar graphs (a-e) presented as mean ± SEM; statistical evaluation of (a-e) performed using one-way ANOVA and Tukey's post hoc multiple comparisons. Non-significant comparisons not identified. *p<0.05, **p<0.01, ***p<0.001, ****p<0.0001.

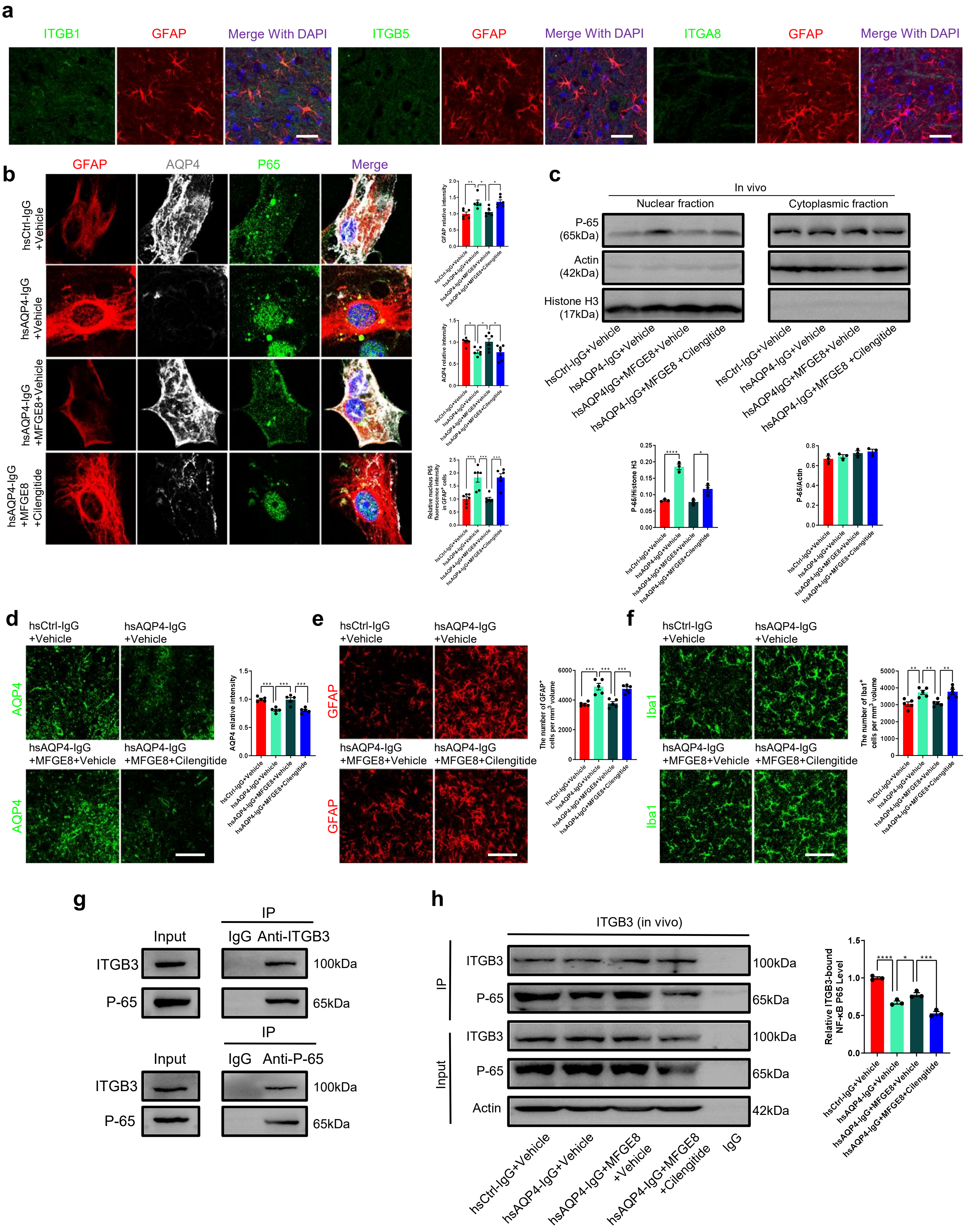
a
ITGB1
GFAP
Merge With DAPI
ITGB5
GFAP
Merge With DAPI
ITGA8
GFAP
Merge With DAPI
b
GFAP
AQP4
P65
Merge
hsCtrl-IgG +Vehicle
hsAQP4-IgG +Vehicle
hsAQP4-IgG +MFGE8+Vehicle
hsAQP4-IgG +MFGE8 +Cilengitide
GFAP relative intensity
AQP4 relative intensity
Relative nucleus P65 fluorescence intensity in GFAP+ cells
c
In vivo
Nuclear fraction
Cytoplasmic fraction
P-65 (65kDa)
Actin (42kDa)
Histone H3 (17kDa)
hsCtrl-IgG+Vehicle
hsAQP4-IgG+Vehicle
hsAQP4IgG+MFGE8+Vehicle
hsAQP4-IgG+MFGE8 +Cilengitide
P-65/Histone H3
P-65/Actin
d
hsCtrl-IgG +Vehicle
hsAQP4-IgG +Vehicle
hsAQP4-IgG +MFGE8+Vehicle
hsAQP4-IgG +MFGE8+Cilengitide
AQP4
AQP4 relative intensity
e
GFAP
The number of GFAP+ cells per mm3 volume
f
Iba1
The number of Iba1+ cells per mm3 volume
g
Input
IP
IgG
Anti-ITGB3
ITGB3
P-65
100kDa
65kDa
Anti-P-65
h
ITGB3 (in vivo)
IP
Input
ITGB3
P-65
Actin
100kDa
65kDa
42kDa
hsCtrl-IgG+Vehicle
hsAQP4-IgG+Vehicle
hsAQP4-IgG+MFGE8 +Vehicle
hsAQP4-IgG+MFGE8 +Cilengitide
IgG
Relative ITGB3-bound NF-κB P65 Level

Supplementary Figure 5. Pharmacological inhibition of integrin αvβ3 reverses the protective role of MFGE8 *in vitro*

(a) Expression of ITGB1, ITGB5, ITGA8, common MFGE8 receptors for immune cells, in ventral horn astrocytes of L4 spinal cord from 8-week-old wildtype mice, assessed by immunostaining of ITGB1 (green) and GFAP (red) (left panels), ITGB5 (green) and GFAP (red) (middle panels), ITGA8 (green) and GFAP (red) (right panels), plus nuclear marker DAPI (blue). Fluorescence signals indicate little colocalization between ITGB1, ITGB5, ITGA8, and astrocyte marker GFAP. Scale bars, 50 μm.

(b) Primary cultures of wildtype mouse astrocytes treated with hsAQP4-IgG (100 ng/mL) and hsCtrl-IgG (100 ng/mL), followed by MFGE8 or MFGE8 with Cilengitide (10 μM) for 4 hours. Primary mouse astrocytes used for immunostaining; representative confocal images (left panels) of GFAP (red), AQP4 (gray), and NF-κB-P65 (green) for indicated groups. Quantification of fluorescence signals (right bar graphs) shows Cilengitide treatment inhibits MFGE8-induced active NF-κB-P65 nuclear accumulation (n=6 experiments/group). Scale bar, 20 μm.

(c) Primary mouse astrocytes treated with hsAQP4-IgG or hsCtrl-IgG, followed by MFGE8 or MFGE8 with Cilengitide for 4 hours; cytoplasmic and nuclear fractions lysed. Lysates subjected to immunoblotting for NF-κB-p65, β-actin, and histone H3 protein levels. Densitometric quantifications (lower panels) reveal Cilengitide-induced active NF-κB-P65 nuclear accumulation compared to MFGE8 treatment (n=3 experiments/group).

(d) Representative confocal images and quantification of AQP4-positive fluorescence signals in spinal cord L4 ventral horn for indicated groups, showing Cilengitide diminishes MFGE8 treatment-associated prevention of AQP4 signal loss (n=5 animals/group). Scale bar, 100 μm.

(e) Representative confocal images and quantification of $GFAP^+$ astrocytes in spinal cord L4 ventral horn for indicated groups, showing Cilengitide diminishes MFGE8 treatment-associated inhibition of astrocyte reactivation (n=5 animals/group). Scale bar, 100 μm.

(f) Representative confocal images and quantification of $Iba1^+$ microglia-positive fluorescence signals in spinal cord L4 ventral horn for indicated groups, showing Cilengitide diminishes MFGE8 treatment-associated inhibition of microglia reactivation (n=5 animals/group). Scale bar, 100 μm.

(g) Primary mouse astrocytes lysed; total protein harvested. Astrocyte lysates subjected to co-IP to assay NF-κB-P65 and ITGB3 interaction alongside IgG controls, revealing ITGB3 interaction with NF-κB-P65 (n=3 experiments/group).

(h) Representative western blot images of in vivo NF-κB-P65 and ITGB3 interaction for indicated groups (left panels). Densitometric quantifications (right bar graph) showing diminished interaction between NF-κB-P65 and ITGB3 in spinal cord L4 of hsAQP4-IgG-treated NMO model mouse compared with hsCtrl-IgG-treated, and enhanced interaction treated with MFGE8. Application of Cilengitide could weaken the role of MFGE8 (n=3 experiments/group).

All data in bar graphs (b-f) presented as mean ± SEM; statistical evaluation of (b-f) performed using one-way ANOVA and Tukey's post hoc multiple comparisons.

Supplementary Figure 6

Uncropped scans of Fig. 1e

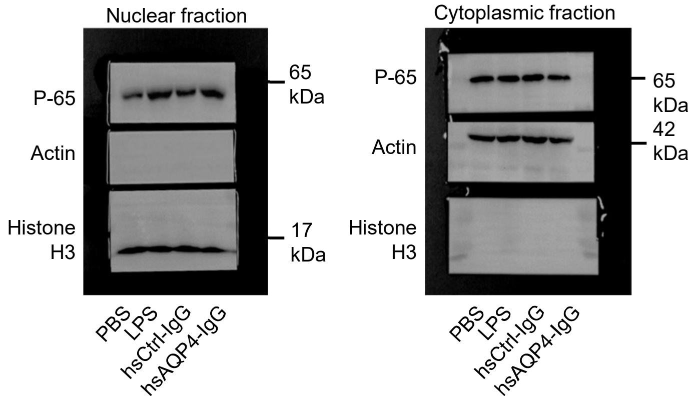

Uncropped scans of Fig. 1i

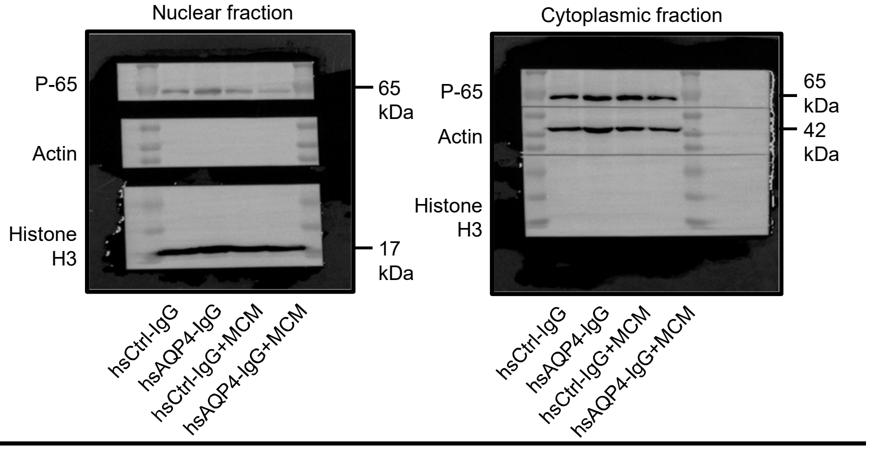

Uncropped scans of Fig. 3k

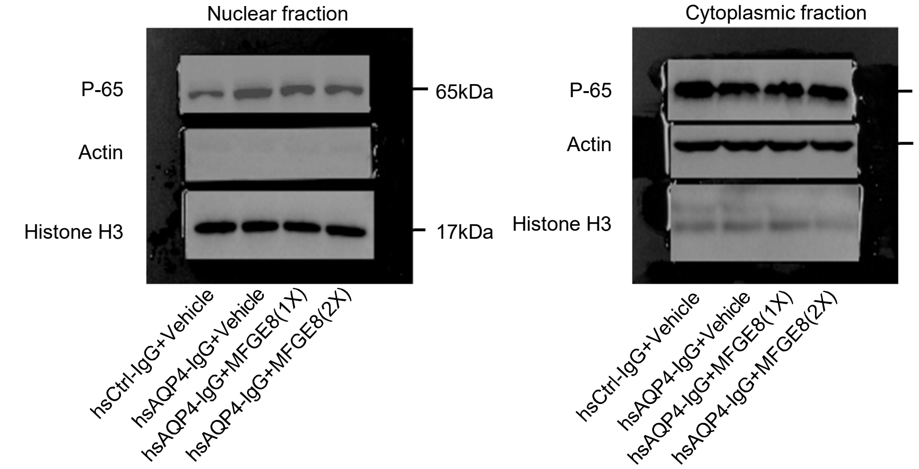

Uncropped scans of Fig. 4a

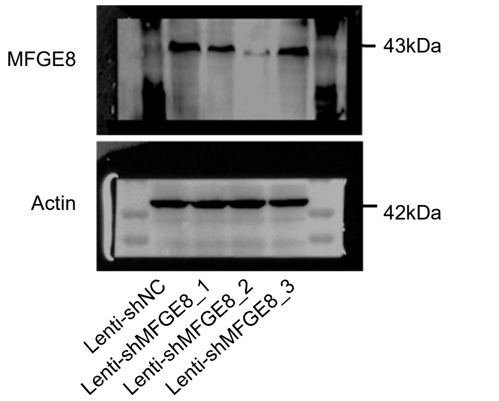

Uncropped scans of Fig. 4g

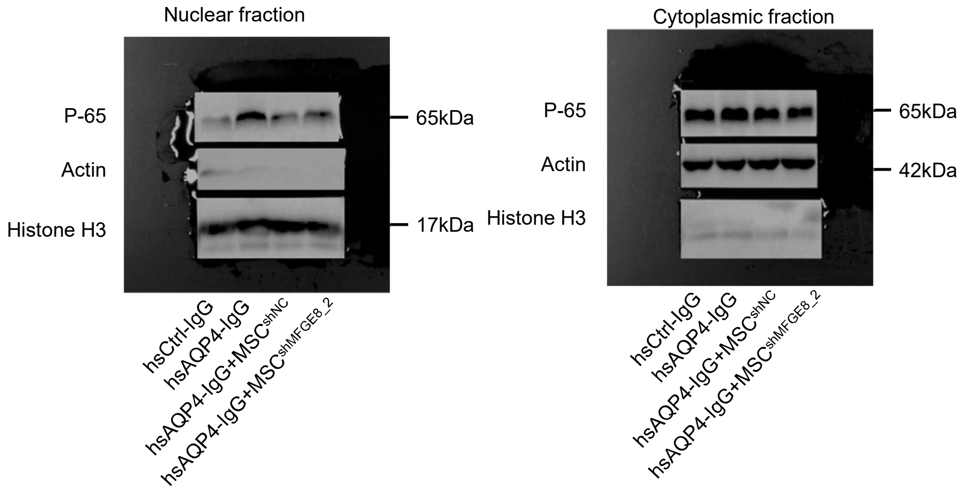

Uncropped scans of Fig. 5j

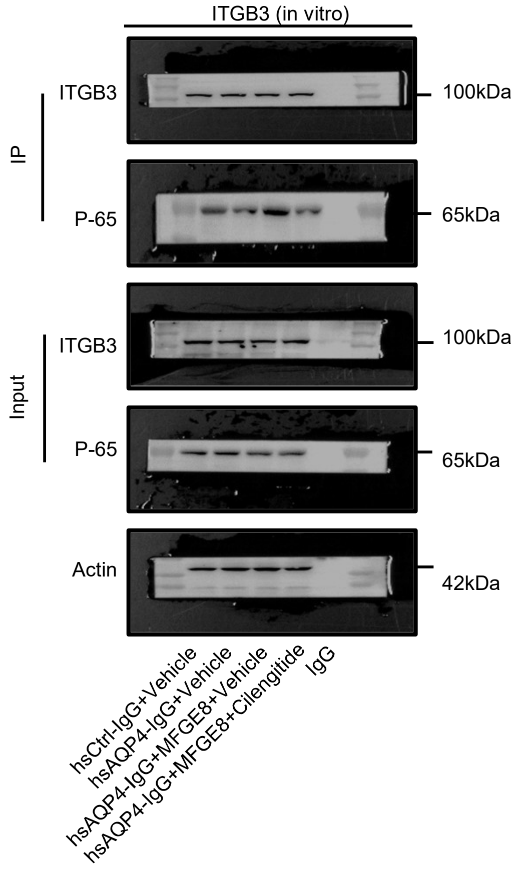

Uncropped scans of Fig. 5g

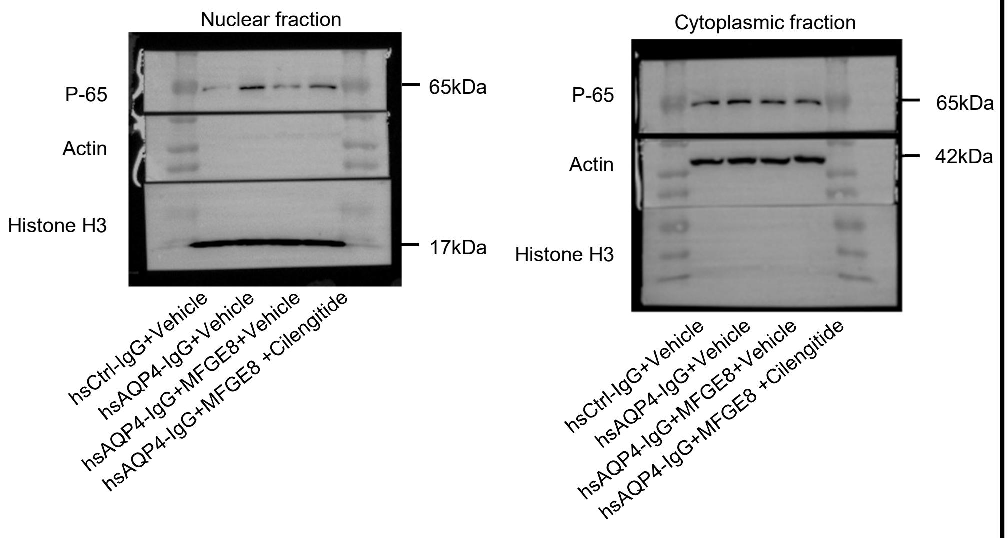

Supplementary Figure 7

Uncropped scans of Supplementary Fig. 2j

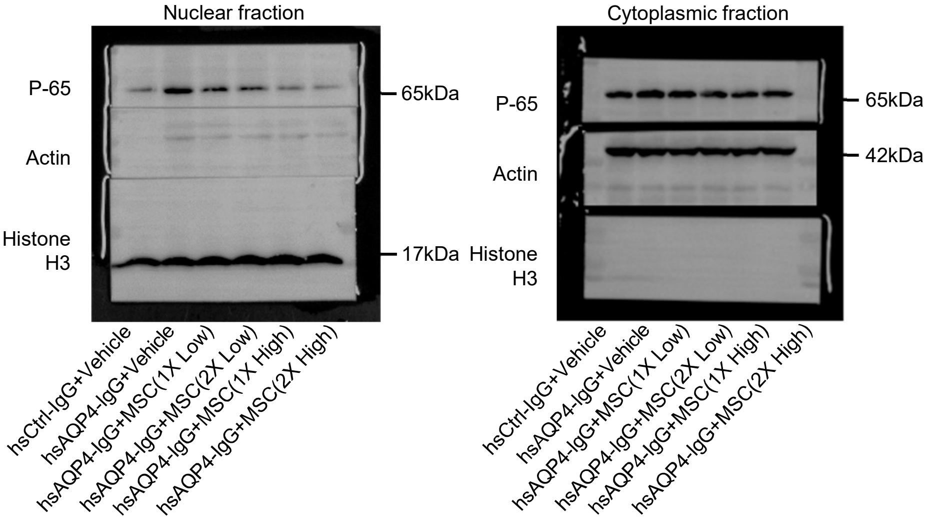

Uncropped scans of Supplementary Fig. 3f

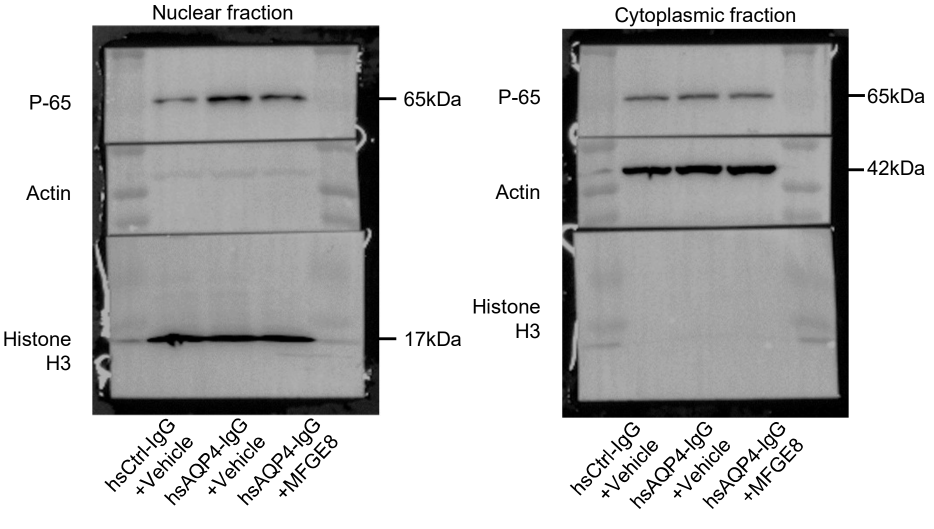

Uncropped scans of Supplementary Fig. 4e

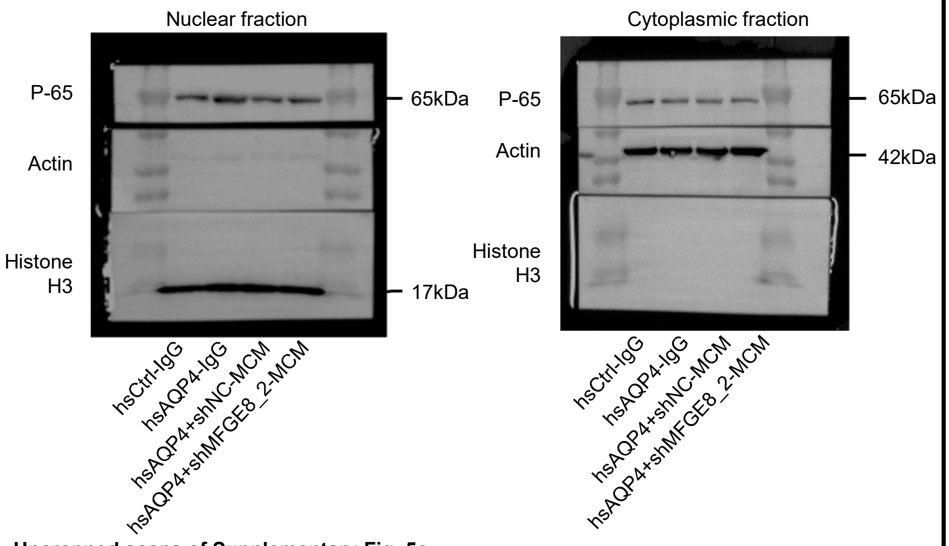

Uncropped scans of Supplementary Fig. 5c

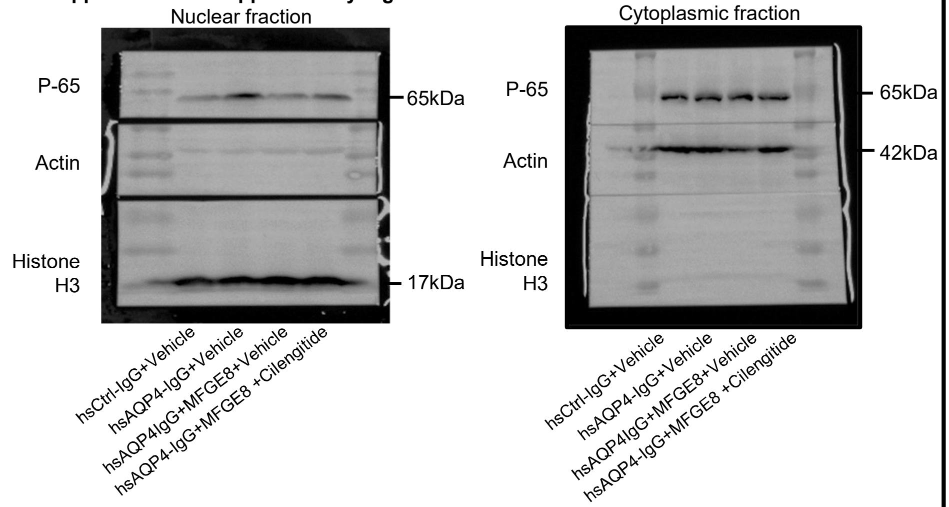

Uncropped scans of Supplementary Fig. 5g

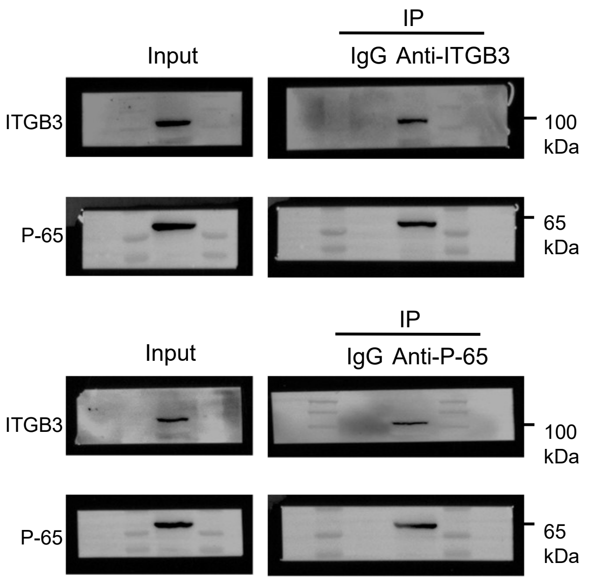

Uncropped scans of Supplementary Fig. 5h

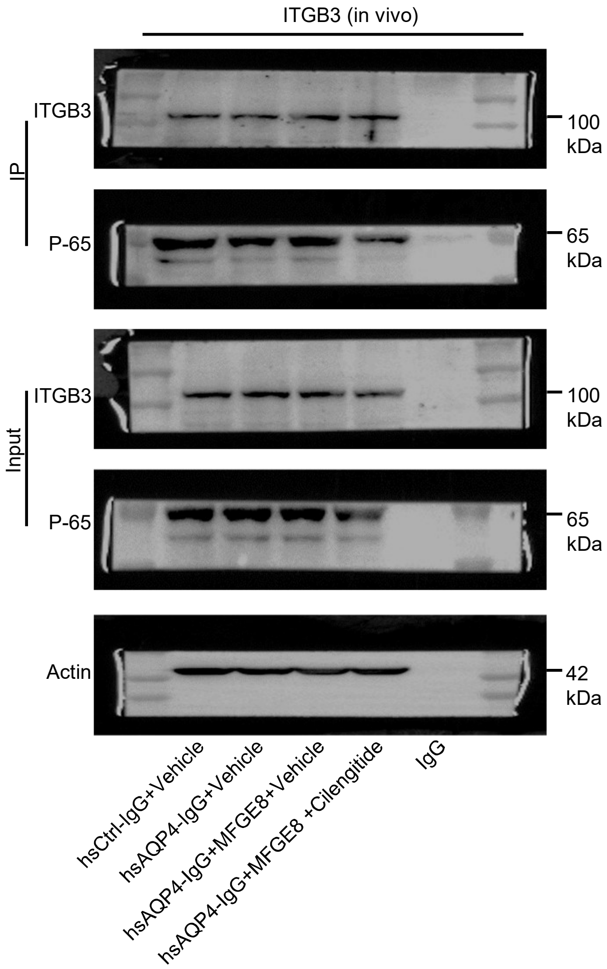

Supplementary Table 1. The summary of previous clinical studies about NF-κB-targeted pro-inflammatory cytokines in NMOSD patients

PMID	Study	Groups	Number	Age (years), mean ± SD	Female/ Male (N)	Disease duration, mean± SD (y)	Mean EDSS scores, mean± SD	Cytokines	Cytokines in serum or CSF (pg/mL)	P-value	Reference DOI
35659638	Wang et al. (2022)	NMOSD	59	42.1 ± 15.3	49/10	16.5 (0-168)	3.8 (0-8.5)	IL-6	Serum:22.7 ± 112.9 (NMOSD); 2.16 ± 0.7 (HC)	*P*<0.001	10.1016/j.neuroscience.2022.05.038
		HC	21	39.1 ± 12.9	20/1	/	/				
30423585	Liu et al. (2016)	NMOSD	31	38.0	20/11	3.0	4.5	IL-1β	CSF:1.71 ± 1.45 (NMOSD); 0.72 ± 0.33 (ONNDs)	*P*<0.001	10.1159/000489561
		ONNDs	22	35.5	14/8	/	/	IL-6	CSF:11.30 ± 19.76 (NMOSD); 5.74 ± 1.74 (ONNDs)	*P*<0.001	
23637915	Matsushita et al. (2013)	NMOSD	27	49.1 ± 11.4	17/3	10.6 ± 9.7	5.6 ± 2.5	CXCL10	CSF: UP (NMOSD)	*P*<0.01	10.1371/journal.pone.0061835
		ONDs	18	46.3 ± 17.5	6/12	/	/	CCL4	CSF: UP (NMOSD)	*P*<0.01	
27401736	Yang et al. (2016)	NMOSD	25	36.80 ± 2.45	23/2	6.04 ± 1.01	3.24 ± 0.35	CXCL5	Serum: UP (NMOSD)	*P*<0.001	10.1186/s12883-016-0622-3
		HC	20	32.30 ± 1.80	15/5	/	/	IL-1β	Serum: UP (NMOSD)	*P*<0.01	
29497397	Tong et al. (2018)	NMOSD	58	39.53 ± 1.57	54/4	56.11, 2-260	3.54 ± 0.21	IL-1β	Serum:UP (NMOSD)	*P*<0.01	10.3389/fneur.2018.00044
		HC	30	33.90 ± 1.64	22/8	/	/	TNF-a	Serum :UP (NMOSD)	*P*<0.05	
32753407	Yandamuri et al. (2020)	NMOSD	29	31.6 ± 16.8	21/8	/	/	CCL3	Serum:128 (NMOSD); 98.6 (HC)	*P*=0.0071	10.1212/NXI.0000000000000852
		HC	11	54.5 ± 17.5	8/3	/	/	IL-6	Serum:3.04 (NMOSD); 1.81 (HC)	*P*=0.0018	
25468778	Li et al. (2015)	NMOSD	35	46.54 ± 13.07	30/5	7.00 ± 7.40 (1–34)	4.53 ± 2.51 (1–9)	TNF-a	Serum:UP (NMOSD)	*P*=0.0015	10.1016/j.jneuroim.2014.11.011
		HC	20	48.40 ± 14.28	18/2	/	/				

Abbreviation: NMO, neuromyelitis optica spectrum disorders. CSF, cerebrospinal fluid. EDSS, expanded disability status scale. ONDs, other neurological diseases. ONNDs, other non-inflammatory neurological diseases.

Supplementary Table 2. The clinical characteristics and demography of health controls and NMO patients enrolled in this study

	HCs	NMO patients	P-value
Age (years)	39.45 ± 7.45	43.20 ± 13.54	0.1108
Men/Women (n)	22/22	4/41	$<$ 0.001
Years of education	11.02 ± 3.19	10.40 ± 6.26	0.5574
AQP4-Ab, positive/negative	-	45/0	-
Disease duration (months)	-	40.96 ± 12.04	-
Mean EDSS scores	-	3.33 ± 1.32	-
Brain lesions (n)	-	37/45	-
Optic neuritis lesions (n)	-	36/45	-
Myelitis lesions (n)	-	45/45	-

Supplementary Table 3. Sequences of the primers used for quantitative real-time PCR

Genes	Forward Primer	Reverse Primer
CCL5	CCTCACCATCCTCACTG	TCTTCTCTGGGTTGGCACAC
CCL7	ATCTCTGCCACGCTTCTGTG	CCTCTTGGGGATCTTTTGTTT
IL-1α	CAGTGAGACCTTCACTGAAG	CTGGAAGTCTGTCATAGAGG
CXCL9	AACGGAGATCAAACCTGCCT	AGATTCAGGGTGCTTGTTGGT
IL-1β	GGCTGCTTCCAAACCTTTGA	GAAGACACGGATTCCATGGT
CCL3	CTGCCCTTGCTGTTCTTCTC	CTTGGACCCAGGTCTCTTTG
CXCL10	CATCCCTGCGAGCCTATCC	CATCTCTGCTCATCATTCTTTTTC
CXCL5	GCCCTACGGTGGAAGTCATA	GTGCATTCCGCTTAGCTTTC
TNF-a	GACTCAAATGGGCTTTCCGA	CCAGCCTCATTCTGAGACAGAG
CCL4	GCCCTCTCTCTCCTCTTGCT	GTCTGCCTCTTTTGGTCAGG
IL-6	GCTTAATTACACATGTTCTCTGGGAAA	CAAGTGCATCATCGTTGTTCATAC
GAPDH	GGGTGTGAACCACGAGAAAT	ACTGTGGTCATGAGCCCTTC
