# Supplementary material for: hUC-MSCs-derived MFGE8 ameliorates locomotor dysfunction via inhibition of ITGB3/ NF-κB signaling in an NMO mouse model
Source: NPJ Regen Med. 2024 Jan 20;9:4. doi: 10.1038/s41536-024-00349-z (PMC10798960; doi:10.1038/s41536-024-00349-z)
Supplement: Supplementary file 2 — reporting-summary [file 41536_2024_349_MOESM2_ESM.pdf]

nature portfolio

Corresponding author(s): Changyong Tang

Last updated by author(s): Jan 7, 2024

Reporting Summary

Nature Portfolio wishes to improve the reproducibility of the work that we publish. This form provides structure for consistency and transparency in reporting. For further information on Nature Portfolio policies, see our Editorial Policies and the Editorial Policy Checklist.

Statistics

For all statistical analyses, confirm that the following items are present in the figure legend, table legend, main text, or Methods section.

n/a	Confirmed	
☐	☒	The exact sample size (*n*) for each experimental group/condition, given as a discrete number and unit of measurement
☐	☒	A statement on whether measurements were taken from distinct samples or whether the same sample was measured repeatedly
☐	☒	The statistical test(s) used AND whether they are one- or two-sided<br>*Only common tests should be described solely by name; describe more complex techniques in the Methods section.*
☐	☒	A description of all covariates tested
☐	☒	A description of any assumptions or corrections, such as tests of normality and adjustment for multiple comparisons
☐	☒	A full description of the statistical parameters including central tendency (e.g. means) or other basic estimates (e.g. regression coefficient) AND variation (e.g. standard deviation) or associated estimates of uncertainty (e.g. confidence intervals)
☒	☐	For null hypothesis testing, the test statistic (e.g. *F*, *t*, *r*) with confidence intervals, effect sizes, degrees of freedom and *P* value noted<br>*Give P values as exact values whenever suitable.*
☒	☐	For Bayesian analysis, information on the choice of priors and Markov chain Monte Carlo settings
☒	☐	For hierarchical and complex designs, identification of the appropriate level for tests and full reporting of outcomes
☒	☐	Estimates of effect sizes (e.g. Cohen's *d*, Pearson's *r*), indicating how they were calculated

Our web collection on statistics for biologists contains articles on many of the points above.

Software and code

Policy information about availability of computer code

Data collection	Leica Application Suite X, XR-FP101, Tanon 5200CE, BioTeck Synergy H1, QuantStudio 5
Data analysis	Leica Application Suite X, XR-FP101, Image J, GraphPad 9

For manuscripts utilizing custom algorithms or software that are central to the research but not yet described in published literature, software must be made available to editors and reviewers. We strongly encourage code deposition in a community repository (e.g. GitHub). See the Nature Portfolio guidelines for submitting code & software for further information.

Data

Policy information about availability of data

All manuscripts must include a data availability statement. This statement should provide the following information, where applicable:

- Accession codes, unique identifiers, or web links for publicly available datasets
- A description of any restrictions on data availability
- For clinical datasets or third party data, please ensure that the statement adheres to our policy

All data collected and analyzed during the current study are available from the corresponding author upon reasonable request.

Research involving human participants, their data, or biological material

Policy information about studies with human participants or human data. See also policy information about sex, gender (identity/presentation), and sexual orientation and race, ethnicity and racism.

Reporting on sex and gender	Not applicable
Reporting on race, ethnicity, or other socially relevant groupings	Not applicable
Population characteristics	Not applicable
Recruitment	Not applicable
Ethics oversight	Not applicable

Note that full information on the approval of the study protocol must also be provided in the manuscript.

Field-specific reporting

Please select the one below that is the best fit for your research. If you are not sure, read the appropriate sections before making your selection.

☒ Life sciences ☐ Behavioural & social sciences ☐ Ecological, evolutionary & environmental sciences

For a reference copy of the document with all sections, see nature.com/documents/nr-reporting-summary-flat.pdf

Life sciences study design

All studies must disclose on these points even when the disclosure is negative.

Sample size	132 mice were used to experiments. For experiments collecting independent values, we used 3-8 distinct samples for each group. These are based on our prior experience to acquire statistical significance. No sample size calculation was used.
Data exclusions	No data were excluded from analysis.
Replication	To acquire independent values from distinct samples, 3-8 replication were used.
Randomization	Mice were randomized prior to allocation to each group.
Blinding	Not applicable

Reporting for specific materials, systems and methods

We require information from authors about some types of materials, experimental systems and methods used in many studies. Here, indicate whether each material, system or method listed is relevant to your study. If you are not sure if a list item applies to your research, read the appropriate section before selecting a response.

Materials & experimental systems

n/a	Involved in the study	
☐	☒	Antibodies
☐	☒	Eukaryotic cell lines
☒	☐	Palaeontology and archaeology
☐	☒	Animals and other organisms
☐	☒	Clinical data
☒	☐	Dual use research of concern
☒	☐	Plants

Methods

n/a	Involved in the study	
☒	☐	ChIP-seq
☐	☒	Flow cytometry
☒	☐	MRI-based neuroimaging

Antibodies

Antibodies used	rabbit anti-AQP4 (1:500, Introvigen, PA5-53234), mouse anti-GFAP (1:1000, CST, 3670S), rabbit anti-Iba1 (1:1000, Wako Chemicals, 019-19741), mouse anti-NeuN (1:500, Abcam, ab104224), rabbit anti-MFGE8 (1:1000, Introvigen, PA5-109955), rabbit anti-integrin-β1 (1:200, Proteintech, 12594-1-AP), rabbit anti-integrin-β3 (1:200, Proteintech, 18309-1-AP), rabbit anti-integrin-β5 (1:200,

	Proteintech, 28543-1-AP), rabbit anti-integrin-α8 (1:200, Santa Cruz Biotechnology, sc-365798), and mouse anti-NF-κB-P65 (1:200, CST,6956). goat anti-mouse 488 (1:1000, Invitrogen), goat anti-mouse 568 (1:1000, Invitrogen), goat anti-mouse 647 (1:1000, Invitrogen), goat anti-rabbit 488 (1:1000, Invitrogen), goat anti-rabbit 568 (1:1000, Invitrogen), goat anti-rabbit 647 (1:1000, Invitrogen), and goat anti-chicken 647 (1:1000, Invitrogen), mouse anti-β-actin (1:1000, HuABio, EM21002), mouse anti-histone H3 (1:1000, HuABio, M1309-1), PE anti-human CD73 (1:100, BioLegend, 344004), APC anti-human CD90 (1:100, BioLegend, 328114), APC anti-human CD34 (1:100, BioLegend, 343608), Alexa Fluor® 488 anti-human CD45 (1:100, BioLegend, 304017), PE anti-human CD11b (1:100, BioLegend, 301306), PE/Cyanine7 anti-human CD19 (1:100, BioLegend, 302216), and PE/Cyanine7 anti-human HLA-DR (1:100, BioLegend, 307616) and Alexa Fluor® 488 anti-human CD105 (1:100, Abcam, ab187575).
Validation	All antibodies were validated by the manufactures.

Eukaryotic cell lines

Policy information about cell lines and Sex and Gender in Research

Cell line source(s)	ATCC, CRL-11268
Authentication	Not applicable
Mycoplasma contamination	Not applicable
Commonly misidentified lines (See ICLAC register)	Not applicable

Animals and other research organisms

Policy information about studies involving animals; ARRIVE guidelines recommended for reporting animal research, and Sex and Gender in Research

Laboratory animals	Wild type C57BL/6 mice (8-12w, 18-24g) were used.
Wild animals	The study did not involve wild animals.
Reporting on sex	Sex was considered in the study design. Female animals were used in each group.
Field-collected samples	The study did not involve samples collected from the field.
Ethics oversight	All procedures and feeding were carried out in accordance with the National Institutes of Health Guide for the Care and Use of Laboratory Animals approved by the Animal Care Committee and the Ethics committee of the Third Affiliated Hospital of Sun Yat-sen University.

Note that full information on the approval of the study protocol must also be provided in the manuscript.

Clinical data

Policy information about clinical studies

All manuscripts should comply with the ICMJE guidelines for publication of clinical research and a completed CONSORT checklist must be included with all submissions.

Clinical trial registration	This study was approved by the ethics committees of the Third Affiliated Hospital of Sun Yat-sen University ([2021]02-405-01)
Study protocol	Not applicable
Data collection	Researchi participants (serum) and data collection were performed at the Third Affiliated hospital of Sun Yat-sen University
Outcomes	The primary outcome of the human cohort was compare the concentration of MFGE8 in the serum of NMOSD and healthy controls. Association between MFGE8 levels and EDSS outcomes were explored.

Plants

Seed stocks	Not applicable
Novel plant genotypes	Not applicable
Authentication	Not applicable

Flow Cytometry

Plots

Confirm that:

☒ The axis labels state the marker and fluorochrome used (e.g. CD4-FITC).

☒ The axis scales are clearly visible. Include numbers along axes only for bottom left plot of group (a 'group' is an analysis of identical markers).

☒ All plots are contour plots with outliers or pseudocolor plots.

☒ A numerical value for number of cells or percentage (with statistics) is provided.

Methodology

Sample preparation	To obtain single cell suspension of hUC-MSCs, Cultured hUC-MSCs which reached 80-90% confluence were treated by 0.25% trypsin-EDTA, The dispersed cells were re-suspended in PBS containing 1% FBS.
Instrument	Cells were acquired on an DxFLEX flow cytometer [Beckman].
Software	Data were analyzed usine CytExpert1.1 software.
Cell population abundance	Sorting was not performed in this study.
Gating strategy	FSC/SSC: forward scatter/sideward scatter.Flow cytometric analysis showed that hUC-MSCs were positive for mesenchymal lineage markers (CD73, CD90, and CD105), negative for hematopoietic and endothelial markers (CD34, CD45, CD11b, CD19), and negative for HLA-DR.

☒ Tick this box to confirm that a figure exemplifying the gating strategy is provided in the Supplementary Information.
